# Smoking Patterns, Quitting Intentions and Associations With Pro‐ and Anti‐Smoking Messages: Insights From the Indonesian GATS Survey (2011 and 2021)

**DOI:** 10.1002/puh2.70202

**Published:** 2026-02-23

**Authors:** Sayyidati Firdausi, I. Nyoman Sutarsa, Putu Ayu Swandewi Astuti, Matthew Kelly

**Affiliations:** ^1^ National Centre for Epidemiology and Population Health Australian National University Canberra Australia; ^2^ School of Medicine and Psychology Australian National University Canberra Australia; ^3^ Department of Public Health and Preventive Medicine Udayana University Sudirman Campus Denpasar Indonesia

**Keywords:** prevention, quitting intention, smoking prevalence, survey research, tobacco advertising

## Abstract

**Introduction:**

Indonesia has one of the highest smoking prevalences globally. Although studies have examined the impact of pro‐ and anti‐tobacco messaging on smoking behaviour and quitting intentions, research on these influences among Indonesian adults remains limited. This study investigates smoking prevalence trends over the past decade and the relationship between tobacco‐related messaging and adult smoking and quitting behaviour in Indonesia.

**Methods:**

This repeated cross‐sectional study analysed secondary data from the Global Adult Tobacco Survey (GATS) Indonesia (2011 and 2021). Participants were adults aged ≥15 years, selected through a multi‐stage stratified cluster sampling. GATS 2021 included 9156 completed interviews, whereas GATS 2011 had 8305 completed interviews. Smoking prevalence was compared using the chi‐square test. Multivariable analysis employed complex samples with multiple logistic regressions to assess associations between tobacco advertising, anti‐smoking messaging, smoking behaviour and quitting intentions.

**Results:**

Male smoking prevalence slightly declined from 67.04% (2011) to 64.71% (2021), whereas female prevalence remained stable (2.66%–2.25%). Exposure to anti‐smoking messages is significantly associated with a lower likelihood of being a current smoker (aOR = 0.84, 95% CI = 0.72–0.99) and is linked to higher odds of quitting attempts (aOR = 1.34, 95% CI = 1.06–1.7) and thoughts of quitting (aOR = 1.52, 95% CI = 1.21–1.92). Exposure to pro‐tobacco messages is not associated with smoking behaviour (aOR = 1.05, 95% CI = 0.87–1.27). Among males only, the exposure to anti‐smoking messages is associated with decreased odds of being current smoker (aOR = 0.82, 95% CI = 0.69–0.98); higher odds of quitting attempts (aOR = 1.28, 95% CI = 1.01–1.64); and higher odds of thoughts of quitting (aOR = 1.54, 95% CI = 1.21–1.95).

**Conclusions:**

Smoking prevalence remains high, with pro‐smoking messages linked to higher smoking rates. Although anti‐smoking messages promote quitting intentions, stronger policies and cessation support are needed to reduce smoking.

## Graphical abstract

Smoking prevalence has changed very little over the last 10 years in Indonesia. However, quitting intentions have increased dramatically and are associated with exposure to anti‐smoking messaging. Smoking cessation support and continued public health campaigns are needed.

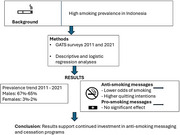

## 1 Introduction

Among Southeast Asian countries, Indonesia has the highest adult smoking rates, with 33.8% of the total population reported as smokers in 2018, and prevalence among males was 62.9%, far above the global and regional prevalence averages [1]. Over the past 10 years, Indonesia's progress in smoking reduction has stalled or even displayed a declining trend [2], with smoking prevalence only declining by a small amount from 34.8% in 2018 to 33.5% in 2021 [3]. Based on Indonesia's Basic National Health Survey 2023, there was a concerning issue regarding the prevalence of youth smokers aged 15–19 years old at 16.7% [4]. Moreover, the national cessation programme has shown limited effectiveness, resulting in low cessation rates of 12.3% among ever smokers in 2015 [5]. This contrast between global progress and Indonesia's persistent or rising smoking burden, particularly among youth, signals gaps in prevention and communication strategies. This lack of progress prevails despite a comprehensive national tobacco control policy that has increased tobacco taxes, established smoke‐free zones, limited advertising and introduced graphic health warnings [3]. Implementation is decentralised into provincial and local government, resulting in inconsistent policies at the national and local levels and gaps between national and regional priorities [6]. Moreover, Indonesia is among the top cigarette markets and exporters worldwide, with the tobacco industry possessing political and economic power [6, 7]. Consequently, tobacco promotions and advertisements are widespread and easily accessible across multiple platforms, including outdoor media, retail outlets, entertainment sponsorships and digital spaces [8]. Given Indonesia's high media penetration and regulatory loopholes, such as weak enforcement of advertising bans and the rapid expansion of social media marketing, exposure to both pro‐ and anti‐tobacco messages is likely to be pervasive and influential.

Prior international studies demonstrated the association between adolescent smoking behaviour and pro‐tobacco advertisements [9, 10, 11]. A 130‐country survey revealed that tobacco marketing at the point of sales was associated with increased smoking experimentation and smoking susceptibility among adolescents [12]. Among youths in Gambia, smoking advertisements increased the odds of being current smokers [10]. Similarly, in Indonesia, exposure to tobacco advertisements, promotions and sponsorships increased the odds of being current smokers and susceptible smokers among youths [11]. On the contrary, among junior and high school students in Indonesia, exposure to pro‐smoking media improved quitting intentions [13].

Anti‐tobacco health promotion programmes in Indonesia enacted by the Ministry of Health [14] include official anti‐smoking promotions aired in mass media [15, 16]. However, the current anti‐smoking campaign faces challenges in terms of persuasive and innovative messages relative to pro‐smoking advertisements [15, 17]. The most recent study in Indonesia shows that exposure to anti‐smoking messages was associated with increased likelihood of being current smokers among high school students [18]. Similarly, in Gambia, adolescents who were exposed to anti‐tobacco messages have increased odds of being current smokers [10]. In contrast, in Vietnam, anti‐smoking advertisement exposure had a positive impact to prevent smoking initiation and trigger thoughts of quitting smoking among youths [17]. Hence, prior studies reveal conflicting information regarding the impact of exposure to anti‐smoking messages on smoking cessation and prevention.

Despite Indonesia having one of the highest smoking prevalence rates globally, there have been only a few in‐depth national studies of smoking patterns and trends over time [2, 3, 4, 5]. Moreover, most research investigating impacts of pro‐ and anti‐tobacco messaging is conducted among adolescents. There remains limited understanding of these exposures and outcomes among the adult population within the Indonesian context [11, 13, 18]. This study investigated these patterns in a nationally representative diverse population sample, aiming to examine associations between pro‐and anti‐smoking messages and adult smoking behaviour and quitting intention using the Global Adult Tobacco Survey (GATS) 2021, as well as comparative analysis with the GATS 2011 survey. We hypothesised that greater exposure to pro‐smoking messages would be associated with higher odds of current smoking and lower quitting intention, whereas greater exposure to anti‐smoking messages would be associated with lower odds of current smoking and higher quitting intention. GATS 2021 remains the most up‐to‐date dataset accessible for assessing smoking behaviour and media exposure. This will provide crucial evidence for tobacco control efforts in Indonesia by providing insights into smoking behaviours and the effectiveness of tobacco regulations and control measures among different demographic groups.

## 2 Methods

### 2.1 Study Design, Sampling and Population

This study uses two approaches. First, a repeated cross‐sectional design investigated changes in prevalence and patterns of smoking behaviour and quitting intentions in Indonesian GATS 2011 and 2021 surveys [19, 20], in response to improvements in tobacco control policies in this period [3]. Second, a cross‐sectional study design using GATS 2021 was utilised to investigate the association between exposure to pro‐ and anti‐smoking messages and adult smoking behaviour and quitting intention. GATS is a national survey for systematic monitoring of tobacco use among adults 15 years and older and is part of the Global Tobacco Surveillance System [21]. Both GATS 2011 and 2021 employed multi‐stage stratified cluster sampling that involved 8305 participants in 2011 (94.3% response rate) and 9156 participants in 2021 (94% response rate) [3]. Multi‐stage cluster sampling involved four stages that are primary sampling units which represents Indonesia's major regions (Sumatra, Java‐Bali, Kalimantan‐Nusa Tenggara and Eastern Indonesia), secondary sampling units, household selection and individual selection [21].

### 2.2 Variables

The exposure variables for this study are pro‐ and anti‐smoking messages. Pro‐smoking message exposure is defined as exposure to cigarette advertisements, promotion and sponsorship in stores, mail, mass media, public places (public transportation included) and social events, including receiving free cigarette samples, coupons and gifts. Participants were classified as experiencing higher exposure (exposure to pro‐smoking media in four or more types) and lower exposure (exposure to three or less pro‐smoking media types). Anti‐smoking messages are mass media that contains health warnings about the danger of smoking. Participants were categorised into exposed (exposure to one or more anti‐smoking media types) and not exposed (not exposed to any anti‐smoking media types). Due to the lower number of avenues for exposure to anti‐smoking messaging, the variable definitions differed from those for pro‐smoking messaging.

The outcome variables of this study are smoking behaviour and quitting intention. Smoking behaviour is defined as tobacco smoking behaviour that includes smoking kretek (cloves) cigarettes, white cigarettes, shisha with tobacco, cigars and pipes. It is categorised as current smokers (daily smokers and occasional smokers) and current non‐smokers (never‐smokers and former smokers). Quitting intention was categorised as (1) attempts to quit smoking in the last 12 months and (2) thoughts of quitting smoking. Participants were classified as ‘yes’ for attempts to quit smoking in the last 12 months if they answered yes to a question regarding any attempts to stop smoking in the last 12 months. Thoughts of quitting smoking were classified as ‘yes’ if respondents reported desire to quit smoking, including within the next month, in the next 12 months and someday. Details of each variable are described in Supporting Information 1.

Analysis co‐variates comprised respondents’ age group (15–24, 25–44, 45–64, ≥65 years), sex (male, female), highest education level (primary school and under, secondary school, high school, college and postgraduate), residence (urban, rural), work status (employed [government employee and non‐government employee], self‐employed [including farmer], non‐labour force [students, homemakers, retired], and unemployed [both able and unable to work]), second‐hand smoking exposure (higher, exposed in three or more places or lower), and knowledge (higher, if the respondent correctly answered seven or more questions about smoking and health, and lower if fewer than seven were answered correctly), smoking level (heavy, 14 cigarettes or more per day, and light 13 or less cigarettes per day), smoking dependency (higher if respondents felt the urge to smoke within 5–60 min after waking up and lower if the urge to smoke occurred more than 60 min after waking up).

### 2.3 Statistical Analysis

This study employed complete case analysis that considers sample weighting with complex sample analysis using Stata/MP 18.0. Descriptive analysis was used to describe the study samples in 2011 and 2021 in terms of socio‐demographics, smoking behaviour (including mean cigarettes smoked per day), exposure to pro‐ and anti‐smoking messaging and quitting intention which is presented in actual sample size and weighted percentage. The proportions of current smokers in 2011 and 2021 were compared across socio‐demographic groupings using chi‐square tests.

Associations between exposure to pro‐ and anti‐smoking messages and smoking behaviour and quitting intention were measured using GATS 2021 data only. Associations were first measured in univariate unadjusted models and results reported using crude odds ratios and 95% confidence intervals. Then we constructed multivariate models with adjusted odds ratios and 95% confidence intervals, adjusted by potential confounders of sex, age group, education level, residence, work status and second‐hand smoke, with smoking level and smoking dependency added when measuring associations with quitting intention because they portray addiction levels that affect quitting intention behaviour. Given that 96.5% of current smokers were male, analyses were then repeated stratified by sex for smoking behaviour outcome, and male samples only were examined for quitting intention outcome.

## 3 Result

### 3.1 Descriptive Statistics

Table 1 portrays changes in current smoking behaviour in 2021 relative to 2011. Overall, there was a small decrease in male smoking prevalence from 67.04% in 2011 to 64.71% in 2021, whereas female smoking prevalence remained stable at 2.66% in 2011 and 2.25% in 2021. The prevalence of current smokers increased among youth aged 15–24 years old, rising from 26.06% in 2011 to 27.86% in 2021. Conversely, older age (45–64 and ≥65 age groups) experienced significant declines around 5% from 2011 to 2021.

**TABLE 1:** Prevalence of current smoking behaviour in Indonesia Global Adult Tobacco Survey (GATS) survey 2021 (*N* = 2877) and 2011 (*N* = 2855).

Variable	Current smokers in GATS 2011 (*N* = 2855)			Current smokers in GATS 2021 (*N* = 2877)			*p* value^a^
	*N*	%	95% CI	*n*	%	95% CI	
**Age group**							
15–24	355	26.06	23.41–28.89	423	27.86	25.18–30.71	<0.01^a^
25–44	1399	37.63	35.61–39.69	1097	37.68	35.45–39.97	0.75
45–64	855	39.38	36.38–42.45	1057	33.90	31.81–36.06	<0.01^a^
≥65	246	31.13	27.51–34.99	300	26.53	23.05–30.33	<0.01^a^
**Sex**							
Male	2720	67.04	64.44–69.54	2776	64.71	62.72–66.65	<0.01^a^
Female	135	2.66	2.02–3.51	101	2.25	1.56–3.23	<0.01^a^
**Education level**							
Primary school and under	1612	37.54	35.56–39.56	1340	34.9	32.94–36.92	<0.01^a^
Secondary school	486	31.9	29.3–34.62	570	33.49	30.72–36.39	<0.01^a^
High school	607	33.74	31.33–36.25	797	35.48	33.22–37.8	<0.01^a^
College and postgrad	150	27.64	23.75–31.89	170	22.21	18.93–25.87	<0.01^a^
**Residence**							
Urban	1307	31.95	29.83–34.14	1221	32.16	30.24–34.14	<0.01^a^
Rural	1548	37.66	35.31–40.06	1656	35.22	33.27–37.23	<0.01^a^
**Work status**							
Employed	1080	46.76	43.84–49.7	840	45.33	42.43–48.26	<0.01^a^
Self‐employed	1454	50.33	46.89–53.76	1545	49.11	46.44–51.78	<0.01^a^
Non‐labour force	144	6.57	5.08–8.45	231	8.18	6.91–9.67	<0.01^a^
Unemployed	175	31.73	27.02–36.84	261	43.23	37.78–48.85	<0.01^a^
**Second‐hand smoke**							
Higher exposure	1063	41.33	38.04–44.7	1318	47.63	45.46–49.82	<0.01^a^
Lower exposure	1792	31.63	29.86–33.46	1559	25.83	24.15–27.58	<0.01^a^
**Attempts to stop smoking in the last 12 months**							
Yes	821	29.11	25.55–32.95	1158	42.68	39.89–45.56	<0.01^a^
No	2032	70.89	67.05–74.45	1716	57.27	54.44–60.11	<0.01^a^
**Thoughts of quitting**							
Yes	1500	48.72	43.04–54.44	1738	63.32	60.02–66.49	<0.01^a^
No	1445	51.28	45.56–56.96	1139	36.68	33.51–39.98	<0.01^a^

	Current smokers (daily smokers only) in GATS 2011 (*n* = 2425)			Current smokers (daily smokers only) in GATS 2021 (*n* = 2332)			
	*n*	%	95% CI	*n*	%	95% CI	
**Smoking dependency**							
Higher dependency	1655	67.71	62.88–72.01	1374	59.06	54.86–63.13	<0.01^a^
Lower dependency	770	32.29	27.84–36.99	956	40.94	36.87–45.14	<0.01^a^
**Smoking level**							
Heavy smoker	1518	62.33	58.23–66.25	811	33.56	30.78–36.47	<0.01^a^
Light smoker	907	37.67	33.75–41.77	1521	66.44	63.53–69.22	<0.01^a^

*Note:* More detailed breakdowns of the variables included in this table are described in Supporting Information 2.

^a^ The *p* values are comparing smoking prevalence in various groups between 2011 and 2021 using chi‐square test.

Looking at different education levels, there is a small change in smoking behaviour in 2011 and 2021. In contrast, a clear trend emerges between those who were actively engaged in the labour force and those who were not. Among those who were actively engaged in the labour force (employed and self‐employed), there was a small decrease in smoking prevalence by approximately 1% meanwhile, among those who were not actively participating in the workforce (non‐labour force and unemployed group), the prevalence of current smokers increased by 1.61% and 11.5%, respectively, during the same period. In the context of residence, the prevalence of urban residents who smoke increased slightly from 31.95% in 2011 to 32.16% in 2021.

Among current smokers, the prevalence of respondents who made attempts to quit smoking in the last 12 months significantly rose from 29.11% in 2011 to 42.7% in 2021. Correspondingly, the prevalence of current smokers who thought of quitting smoking increased from 48.72% in 2011 to 63.32% in 2021. It is also evident that there was an increasing trend of quitting intention behaviour based on all respondents’ socio‐demographic characteristics (see Supporting Information 2 and 3). Daily smokers with higher smoking dependency recorded a significant decline from 67.71% in 2011 to 59.06% in 2021. The prevalence of heavy smokers also saw a major decrease from 62.33% in 2011 to 33.56% in 2021. Light smokers increased from 37.67% in 2011 to 66.44% in 2021.

In Table 2, it is evident that most Indonesian smokers were males. Among males, the prevalence of current smokers stands at 64.71%. Conversely, among females, only 2.25% are current smokers. Regarding the exposure variable, among respondents who were exposed to anti‐smoking messages, 34.61% were current smokers, of whom 47.46% had attempted to quit smoking in the last 12 months and 68.96% thought to quit smoking someday. Meanwhile, among those who were more exposed to pro‐smoking messages, 39.16% were identified as current smokers, of whom 49.81% had attempted to quit smoking in the last 12 months and 70.37% thought to quit smoking someday.

**TABLE 2:** Socio‐demographic and exposure characteristics of study sample and smoking behaviour among Indonesian adults in 2021 (row percentage, weighted, *N* = 2877).

Variable	Smoking behaviour (current smokers) (*N* = 2877)			Attempts to quit smoking in the last 12 months (Yes) (*N* = 2877)				Thoughts of quitting (Yes) (*N* = 2877)	
	*n*	%	95% CI	*n*	%	95% CI	*n*	%	95% CI
**Anti‐smoking messages**									
Not exposed	1155	31.7	29.6–33.86	384	34.64	30.41–39.12	583	53.77	48.87–58.6
Exposed	1722	34.61	32.92–36.33	774	47.46	44.32–50.63	1155	68.96	65.48–72.24
**Pro‐smoking messages**									
Higher exposure	1093	39.16	36.8–41.57	522	49.81	45.76–53.86	766	70.37	66.5–73.96
Lower exposure	1784	30.11	28.3–31.99	636	37.25	33.82–40.81	972	57.92	53.55–62.16
**Age group**									
15–24	423	27.86	25.18–30.71	219	52.3	44.8–59.63	280	65.87	59.63–71.59
25–44	1097	37.68	35.45–39.97	450	42.02	38.26–45.88	709	65.79	61.55–69.8
45–64	1057	33.90	31.81–36.06	388	39.46	35.85–43.18	617	62.12	58.15–65.93
≥65	300	26.53	23.05–30.33	101	36.41	29.38–44.07	132	46.33	38.84–53.99
**Sex**									
Male	2776	64.71	62.72–66.65	1112	42.62	39.89–45.4	1674	63.32	60.09–66.44
Female	101	2.25	1.56–3.23	46	44.98	31.46–59.29	64	63.19	49.47–75.06
**Education level**									
Primary school and under	1340	34.9	32.94–36.92	463	36.96	33.61–40.43	688	53.44	49.13–57.69
Secondary school	570	33.49	30.72–36.39	242	43.97	39.37–48.67	357	63.94	58.25–69.25
High school	797	35.48	33.22–37.8	371	47.95	44.11–51.82	562	71.87	66.85–76.4
College and postgrad	170	22.21	18.93–25.87	82	47.73	38.44–57.18	131	79.01	71–85.26
**Residence**									
Urban	1221	32.16	30.24–34.14	553	47.07	43.59–50.58	820	69.4	65.15–73.34
Rural	1656	35.22	33.27–37.23	605	37.32	32.97–41.89	918	55.85	50.67–60.91
**Work status**									
Employed	840	45.33	42.43–48.26	353	43.98	39.73–48.33	573	69.76	65.41–73.79
Self‐employed	1545	49.11	46.44–51.78	551	38.14	34.69–41.73	864	59.07	55.29–62.75
Non‐labour force	231	8.18	6.91–9.67	125	53.67	45.06–62.07	153	66.02	57.66–73.49
Unemployed	261	43.23	37.78–48.85	129	52.02	44.3–59.65	148	60.99	51.46–69.76
**Second‐hand smoke**									
Higher exposure	1318	47.63	45.46–49.82	560	44.94	41.18–48.76	855	66.96	63.14–70.56
Lower exposure	1559	25.83	24.15–27.58	598	40.47	36.84–44.2	883	59.7	55.23–64.02
**Attempts to stop smoking in the last 12 months**									
Yes	1158	42.68	39.89–45.56				891	78.75	75.14–81.95
No	1716	57.27	54.44–60.11				845	51.8	47.78–55.8
**Thoughts of quitting**									
Yes	1738	63.32	60.02–66.49	891	53.11	49.73–56.46			
No	1139	36.68	33.51–39.98	267	24.73	21.31–28.5			

	Current smokers (daily smokers only)in GATS 2021 (*n*)			Current smokers (daily Smokers only) in GATS 2021 (*n*)				Current smokers (daily smokers only) in GATS 2021 (*N*)	
	*n*	%	95% CI	n	%	95% CI	*n*	%	95% CI
**Smoking dependency**									
Higher dependency	1374	59.06	54.86–63.13	437	33.49	29.68–37.54	700	53.85	49.49–58.16
Lower dependency	956	40.94	36.87–45.14	396	44.23	39.6–48.96	640	69.4	64.4–73.98
**Smoking level**									
Heavy smoker	811	33.56	30.78–36.47	265	34.85	30.76–39.18	417	52.7	47.86–57.5
Light smoker	1521	66.44	63.53–69.22	568	39.44	36.05–42.94	923	63.95	59.74–67.97

*Note:* More detailed breakdowns of the variables included in this table are described in Tables S2–S4.

### 3.2 Multivariable Analysis

In fully adjusted models (Table 3), exposure to anti‐smoking messages was associated with lower odds of adults smoking (aOR = 0.84, 95% CI = 0.72–0.99) and higher odds of both attempting to quit in the past 12 months (aOR = 1.34, 95% CI = 1.06–1.70) and thinking about quitting (aOR = 1.52, 95% CI = 1.21–1.93). The significant association between exposure to pro‐smoking messaging and likelihood of smoking became non‐significant after adjusting for confounders, although those who were exposed to higher pro‐smoking messages had 30% higher odds of attempts to quit smoking in the last 12 months (aOR = 1.3, 95% CI = 1.01–1.67). Sex was strongly associated with smoking with men having substantially higher odds of smoking than women (aOR = 58.41, 95% CI = 40.88–83.46) and insufficient evidence to prove between the exposure to higher pro‐smoking messages with thoughts of quitting (aOR = 1.08, 95% CI 0.83–1.42).

**TABLE 3:** Crude and adjusted association of the exposure to anti‐smoking messages and pro‐smoking messages and smoking behaviour, attempts to quit smoking in the last 12 months and thoughts of quitting smoking in 2021.

Variable	Smoking behaviour				Attempts to quit in the last 12 months				Thoughts of quitting smoking			
	Crude OR 95% CI	*p* value	Adj OR 95% CI	*p* value	Crude OR 95%CI	*p* value	Adj OR 95% CI	*p* value	Crude OR 95% CI	*p* value	Adj OR 95% CI	*p* value
**Anti‐smoking messages**												
Not exposed	Ref	Ref	Ref	Ref	Ref	Ref	Ref	Ref	Ref	Ref	Ref	Ref
Exposed	1.14 (1.02–1.28)	0.03^*^	0.84 (0.72–0.99)	0.04^*^	1.71 (1.37–2.13)	<0.01^*^	1.34 (1.06–1.70)	<0.01^*^	**1.91 (1.54–2.37)** ^*^	<0.01	1.52 (1.21–1.93)	<0.01^*^
**Pro‐smoking messages**												
Higher exposure	1.49 (1.31–1.71)	<0.01^*^	1.05 (0.87–1.27)	0.61	1.67 (1.35–2.07)	<0.01^*^	**1.30 (1.01–1.67)**	**0.05** ^*^	1.73 (1.37–2.17)	<0.01	1.09 (0.83–1.44)	0.51
Lower exposure	Ref	Ref	Ref	Ref	Ref	Ref	Ref	Ref	Ref	Ref	Ref	Ref
**Sex**												
Male	79.54 (54.79–115.48)	<0.01^*^	58.41 (40.88–83.46)	<0.01^*^	0.91 (0.52–1.58)	0.73	1.26 (0.62–2.53)	0.52			0.55 (0.27–1.13)	0.1
Female	Ref	Ref	Ref	Ref	Ref	Ref	Ref	Ref	Ref	Ref	Ref	Ref
**Age**												
15–24	1.07 (0.85–1.35)	0.56	1.58 (1.15–2.19)	<0.01^*^	1.98 (1.28–3.05)^*^	<0.01^*^	1.47 (0.81–2.67)	0.2	1.33 (0.89–1.98)^*^	0.16	1.24 (0.92–1.67)	0.15
25–44	1.67 (1.36–2.06)	0.00^*^	2.34 (1.80–3.05)	<0.01^*^	1.37 (1.03–1.84)^*^	0.03^*^	1.17 (0.7303 1.88)	0.51	1.55 (1.16–2.07)^*^	<0.01^*^	1.67 (1.24–2.25)	<0.01^*^
45–64	1.42 (1.16–1.73)	0.00	1.55 (1.19–2.03)	<0.01^*^	0.97 (0.78–1.21)	0.81	1.14 (0.77–1.70)	0.51	1.39 (1.12–1.73)^*^	<0.01^*^	2.92 (1.74–4.91)	
≥65	Ref	Ref	Ref	Ref	Ref	Ref	Ref	Ref	Ref	Ref	Ref	Ref
**Education level**												
Primary school and under	Ref	Ref	Ref	Ref	Ref	Ref	Ref	Ref	Ref	Ref	Ref	Ref
Secondary school	0.94 (0.81–1.09)	0.41	0.81 (0.65–0.99)	<0.01*	1.33 (1.11–1.60)	<0.01*	1.01 (0.80–1.30)	0.90	1.54 (1.20–1.99)^*^	<0.01	1.24 (0.92–1.67)	0.15
High school	1.03 (0.92–1.15)	0.66	0.55 (0.46–0.66)	<0.01^*^	1.57 (1.29–1.91)	<0.01^*^	1.01 (0.77–1.33)	0.93	2.23 (1.72–2.88)^*^	<0.01	1.67 (1.24–2.25)	<0.01^*^
College and postgrad	0.53 (0.43–0.66)	<0.01^*^	0.23 (0.18–0.31)	<0.01^*^	1.55 (1.05–2.28)	0.03^*^	1.03 (0.66–1.62)	0.89	3.28 (2.07–5.20)^*^	<0.01	2.92 (1.74–4.91)	<0.01^*^
**Residence**												
Urban	Ref	Ref	Ref	Ref	Ref	Ref	Ref	Ref	Ref	Ref	Ref	Ref
Rural	1.15 (1.01–1.3)	0.03^*^	1.08 (0.90–1.30)	0.4	0.67 (0.53–0.85)	<0.01^*^	0.83 (0.63–1.1)	0.2	0.56 (0.42–0.74)^*^	<0.01	0.82 (0.6–1.12)	0.2
**Work status**												
Employed	Ref	Ref	Ref	Ref	Ref	Ref	Ref	Ref	Ref	Ref	Ref	Ref
Self‐employed	1.16 (0.99–1.37)	0.06	1.09 (0.91–1.30)	0.33	0.79 (0.63–0.98)	0.03^*^	1.02 (0.8–1.30)	0.9	0.63 (0.50–0.79)^*^	<0.01	0.95 (0.75–1.21)	0.68
Non‐labour force	0.11 (0.09–0.13)	<0.01^*^	0.325 (0.25–0.43)	<0.01^*^	1.48 (1.03–2.11)	0.03^*^	1.01 (0.77–1.33)	0.93	0.84 (0.58–1.22)	0.37	0.85 (0.52–1.39)	0.5
Unemployed	0.92 (0.71–1.18)	0.51	1.15 (0.87–1.52)	0.31	1.38 (0.97–1.96)	0.07	1.03 (0.66–1.62)	0.89	0.68 (0.45–1.02)	0.86	0.85 (0.54–1.33)	0.47
**Second‐hand smoke**												
Higher	2.61 (2.31–2.95)	<0.01^*^	1.73 (1.44–2.08)	<0.01^*^	1.2 (0.98–1.47)	0.08	0.99 (0.78–1.26)	0.93	1.37 (1.11–1.69)^*^	<0.01	1.17 (0.91–1.5)	0.21
Lower	Ref		Ref	Ref	Ref	Ref	Ref	Ref	Ref	Ref	Ref	Ref
**Thoughts of quitting**												
Yes					3.45 (2.78–4.27)	<0.01^*^	2.77 (2.18–3.51)	<0.01^*^				
No					Ref	Ref	Ref	Ref				
**Attempt to quit in the last 12 months**												
Yes									3.45 (2.78–4.27)	<0.01	2.77 (2.19–3.52)	<0.01^*^
No									Ref	Ref	Ref	Ref
**Smoking dependency**												
Higher					Ref		Ref					
Lower					1.58 (1.21–2.04)^*^	<0.01^*^	1.34 (1.04–1.73)	0.02^*^	1.94 (1.49–2.54)^*^	<0.01	1.57 (1.21–2.03)	<0.01^*^
**Smoking level**												
Heavy					Ref	Ref	Ref	Ref	Ref	Ref	Ref	Ref
Light					1.22 (0.99–1.49)	0.06	0.98 (0.77–1.25)	0.86	1.59 (1.26–2.00)^*^	<0.01	1.46 (1.13–1.89)	<0.01^*^

*Note:* Models adjusted for sex, age, education, residence, work status and second‐hand smoke exposure.

*p < 0.05.

Relative to the elderly (65 years and above), adults of all age groups had higher odds of being smokers in adjusted models. Respondents who were more exposed to others smoking at home and in public areas have 73% higher odds of being current smokers (aOR = 1.73, 95% CI = 1.44–2.08) relative to those who were less exposed to second‐hand smoke. In terms of education level, relative to primary school and under, adults who finished their education in secondary school (aOR = 0.81, 95% CI = 0.65–0.99), high school and above (aOR high school = 0.73, 95% CI = 0.63–0.86; aOR college/postgrad = 0.25, 95% CI = 0.19–0.31) were less likely to smoke. Meanwhile, attempts to quit smoking in the last 12 months are associated with thoughts of quitting (aOR = 2.76, 95% CI = 2.17–3.50), and lower smoking dependency (aOR = 1.34, 95% CI = 1.04–1.73). Thoughts of quitting are characterised by 45–64 age group (aOR = 1.79, 95% CI = 1.22–2.64), education level (aOR high school = 1.65, 95% CI = 1.22–2.22; aOR college/postgrad = 2.85, 95% CI = 1.72–4.91), lower smoking dependency (aOR = 1.57, 95% CI = 1.21–2.03), light smokers (aOR = 1.44, 95% CI = 1.12–1.86), and those who attempted to stop smoking in the last 12 month (aOR = 1.46, 95% CI = 1.13–1.9).

### 3.3 Subgroup Analysis

Table 4 reveals that among males only, the exposure to anti‐smoking messages is a protective factor of being current smoker (aOR = 0.82, 95% CI = 0.69–98). There is not enough evidence to prove the association of pro‐smoking messages (aOR male = 1.15, 95% CI = 0.93) with male smoking behaviour. Among male respondents only, current smoking is characterised by age group, education level and second‐hand smoking. Adult male respondents (15 years old and older) have higher odds of being current smokers (aOR 15–24 = 1.85, 95% CI = 1.35–2.56; aOR 25–44 = 2.63, 95% CI = 1.99–3.47; aOR 45–64 = 1.53, 95% CI (1.16–2.02). Those who were more exposed to second‐hand smoke have higher odds of being current smokers (aOR = 1.70, 95% CI = 1.41–2.05). Meanwhile, having higher education (high school and above) is protective against being current smokers (aOR high school = 0.54, 95% CI = 0.44–0.66; aOR college/postgrad = 0.24, 95% CI = 0.18–0.32).

**TABLE 4:** Association of the exposure to anti‐smoking messages and pro‐smoking messages and current smoking behaviour, attempts to quit smoking in the last 12 months and thoughts of quitting among males only.

	Stratified OR of male being current smokers (95% CI)				Stratified OR for male who tried to stop smoking in the last 12 months				Stratified OR for male who thought to stop smoking			
Variable	Crude OR (95% CI)	*p* value	Adj OR (95% CI)	*p* value	Crude OR (95% CI)	*p* value	Adj OR (95% CI)	*p* value	Crude OR (95% CI)	*p* value	Adj OR (95% CI)	*p* value
**Anti‐smoking messages**												
Not exposed	Ref	Ref	Ref		Ref	Ref	Ref	Ref	Ref	Ref	Ref	Ref
Exposed	0.85 (0.73–1.00)	0.05^*^	0.82 (0.69–0.98)	0.03^*^	1.64 (1.32–2.04)	<0.01^*^	1.28 (1.01–1.64)	0.04^*^	1.91 (1.55–2.35)	<0.01^*^	1.54 (1.21–1.95)^*^	<0.01^*^
**Pro‐smoking messages**												
Higher	0.99 (0.83–1.17)	0.89	1.10 (0.90–1.34)	0.36	1.68 (1.35–2.09)	<0.01^*^	1.34 (1.03–1.74)^*^	0.03^*^	1.73 (1.37–2.18)	<0.01^*^	1.08 (0.82–1.42)	0.57
Lower	Ref	Ref	Ref	Ref	Ref	Ref	Ref	Ref	Ref	Ref	Ref	Ref
**Age**												
15–24	0.52 (0.40–0.67)	<0.01^*^	1.85 (1.35–2.56)^*^	<0.01^*^	1.98 (1.26–3.09)	<0.01^*^	1.42 (0.79–2.52)	0.24	1.39 (0.93–2.08)	0.11	1.37 (0.82–2.27)	0.22
25–44	1.30 (1.06–1.60)	0.01	2.63 (1.99–3.47)^*^	<0.01^*^	1.38 (1.02–1.85)	0.03^*^	1.05 (0.66–1.65)	0.84	1.61 (1.20–2.14)	<0.01^*^	1.60 (1.01–2.55)	0.05^*^
45–64	1.71 (1.44–2.03)	<0.01^*^	1.53 (1.16–2.02)^*^	<0.01^*^	0.97 (0.77–1.23)	0.82	1.05 (0.70–1.58)	0.80	1.45 (1.17–1.81)		1.9 (1.29–2.81)	<0.01^*^
≥65	Ref	Ref	Ref	Ref	Ref	Ref	Ref	Ref	Ref	Ref	Ref	Ref
**Education level**												
Primary school and under	Ref	Ref	Ref	Ref	Ref	Ref	Ref	Ref	Ref	Ref	Ref	Ref
Secondary school	0.67 (0.54–0.83)	<0.01^*^	0.83 (0.66–1.04)	0.1	1.30 (1.08–1.57)	<0.01^*^	1.05 (0.82–1.34)	0.39	1.59 (1.24–2.04)	<0.01^*^	1.32 (0.98–1.76)	0.06
High school	0.65 (0.54–0.77)	<0.01^*^	0.54 (0.44–0.66)^*^	<0.01^*^	1.52 (1.24–1.86)	<0.01^*^	1.01 (0.77–1.33)	0.92	2.24 (1.74–2.9)	<0.01^*^	1.72 (1.28–2.3)^*^	<0.01^*^
College and postgrad	0.32 (0.24–0.42)		0.24 (0.18–0.32)^*^	<0.01^*^	1.56 (1.06–2.29)	0.02^*^	1.08 (0.69–1.69)	0.74	3.37 (2.12–5.35)	<0.01^*^	3.08 (1.83–5.18)^*^	<0.01^*^
**Residence**												
Urban	0.79 (0.66 0.94)	<0.01^*^	0.92 (0.77–1.11)	0.84	1.45 (1.15–1.83)	<0.01^*^	1.167 (0.88–1.54)	1.11	1.76 (1.33–2.33)	<0.01^*^	1.22 (0.89–1.67)	0.21
Rural	Ref	Ref	Ref	Ref	Ref	Ref	Ref	Ref	Ref	Ref	Ref	Ref
**Work status**												
Employed	Ref	Ref	Ref	Ref	Ref	Ref	Ref	Ref	Ref	Ref	Ref	Ref
Self‐employed	1.31 (1.09–1.56)	<0.01^*^	1.1 (0.93–1.33)	0.26	0.79 (0.63–0.98)	0.03^*^	0.94 (0.71–1.26)	0.69	0.63 (0.50–0.79)	<0.01^*^	0.96 (0.75–1.21)	0.70
Non‐Labour force	0.28 (0.22 0.36)	<0.01^*^	0.26 (0.19–0.36)^*^	<0.01^*^	1.48 (0.99–2.2)	0.06	1.15 (0.62–2.14)	0.65	0.83 (0.56–1.24)	0.37	0.79 (0.46 1.35)	0.38
Unemployed	1.08 (0.81–1.44)	<0.01^*^	1.15 (0.86–1.54)	0.34	1.38 (0.97–1.99)	0.08	1.34 (0.85–2.10)	0.21	0.67 (0.44–1.00)	0.05^*^	0.78 (0.46–1.32)	0.34
**Second‐hand smoke**												
Higher	1.51 (1.28–1.79)	<0.01^*^	1.70 (1.41–2.05)^*^	<0.01^*^	1.17 (0.96–1.43)	0.12	0.99 (0.78–1.25)	0.93	1.34 (1.1–1.65)	<0.01^*^	1.16 (0.91–1.48)	0.23
Lower	Ref	Ref	Ref	Ref	Ref	Ref	Ref	Ref	Ref	Ref	Ref	Ref
**Thoughts of quitting**												
Yes					3.32 (2.67–4.12)	<0.01^*^	2.71 (2.12–3.47)^*^	<0.01^*^				
No					Ref	Ref	Ref	Ref				
**Attempts to quit smoking in the last 12 months**												
Yes									3.32 (2.67–4.11)	<0.01^*^	2.72 (2.13–3.47)^*^	<0.01^*^
No									Ref	Ref	Ref	Ref
**Smoking dependency**												
Higher					Ref	Ref	Ref	Ref	Ref	Ref	Ref	Ref
Lower					1.6 (1.24–2.06)	<0.01^*^	1.35 (1.04–1.73)^*^	0.02^*^	1.96 (1.49–2.58)	<0.01^*^	1.58 (1.20–2.07)^*^	<0.01^*^
**Smoking level**												
Heavy					Ref	Ref	Ref	Ref	Ref	Ref	Ref	Ref
Light					1.24 (1.00–1.53)^*^	0.05^*^	0.99 (0.77–1.27)	0.92	1.58 (1.24–2.00)	<0.01^*^	1.44 (1.11–1.87)^*^	<0.01^*^

*p < 0.05.

Among males only the exposure to both anti‐ and pro‐smoking messages is associated with quitting attempts in the last 12 months (aOR anti‐smoking messages 1.28, 95% CI = 1.01–1.64; aOR higher exposure to pro‐smoking messages 1.34, 95% CI = 1.03–1.74). Attempts to quit smoking in this subgroup is characterised by thoughts of quitting (aOR 2.71, 95% CI = 2.12–3.47) and lower smoking dependency (aOR 1.35, 95% CI = 1.04–1.73).

Among male respondents, the exposure to anti‐smoking messages is associated with thoughts of quitting (aOR = 1.54, 95% CI = 1.21–1.95). There is not enough evidence to prove the association between pro‐smoking messages and thoughts of quitting among males only respondents (aOR = 1.08, 95% CI = 0.82–1.42). In this subgroup, thoughts of quitting are characterised by adult age group (aOR 25–44 = 1.60, 95% CI = 1.01–2.55; aOR 45–64 = 1.9, 95% CI = 1.29–2.81); higher education level (aOR high school = 1.72, 95% CI = 1.28–2.3; aOR college and postgrad = 3.08, 95% CI = 1.83–5.18); attempts to quit in the last 12 months (aOR = 2.72, 95% CI = 2.13–3.47); lower smoking dependency (aOR = 1.58, 95% CI = 1.20–2.07) and light smoker (aOR = 1.44, 95% CI = 1.11–1.87).

### 3.4 Collinearity Test

Table 5 shows that there was no strong association between independent variables in the regression model in which the tolerance value of all variables is greater than 0.1 whereas the VIF value of all variables is smaller than 10.

**TABLE 5:** Results for the collinearity for each outcome variable (smoking behaviour, attempts to quit in the last 12 month and thoughts of quitting).

Variable	Smoking behaviour		Attempts to quit in the last 12 months		Thoughts of quitting	
	Tolerance	VIF	Tolerance	VIF	Tolerance	VIF
Anti‐smoking messages	0.803760	1.24	0.812702	1.23	0.816203	1.23
Pro‐smoking messages	0.749795	1.33	0.730087	1.37	0.729393	1.37
Age	0.853499	1.17	0.817219	1.22	0.815075	1.23
Education level	0.897629	1.11	0.768678	1.30	0.774290	1.29
Residence	0.944208	1.06	0.900475	1.11	0.900350	1.11
Work status	0.951771	1.05	0.976129	1.02	0.975462	1.03
Second‐hand smoke	0.859248	1.16	0.842615	1.19	0.842888	1.19
Knowledge about smoking harm	0.879594	1.14	0.877159	1.14	0.908620	1.10
Thoughts of quitting			0.873668	1.14		
Attempts to quit smoking in the last 12 months					0.953129	1.05
Smoking dependency			0.910818	1.10	0.918205	1.09
Smoking level			0.916486	1.09	0.918747	1.09

## 4 Discussion

This study provides important insights into changes in smoking behaviour in Indonesia between 2011 and 2021, and the association between exposure to pro‐smoking and anti‐smoking messages with smoking behaviour and quitting intentions in 2021. A key finding is that although smoking prevalence has only slightly decreased over the last decade, it has increased among youths aged 15–24. Quitting intention prevalence has increased significantly and is influenced significantly by anti‐smoking messaging and media. This is crucial for understanding tobacco control targets in one of the world's heaviest smoking nations.

Between 2011 and 2021, smoking prevalence among men remained high, whereas women's smoking prevalence has remained low. This study's finding of an increase in smoking prevalence among younger Indonesians contrasts with the findings of the periodic Indonesian National Health Survey, which reported a declining trend in youth smoking (aged 15–24) from 52.4% in 2013 to 47.1% in 2023 [4]. This discrepancy may be attributed to differences in timeline and methodology, as the National Health Survey relies on a larger sample size, whereas GATS employs a weighted sample calculation to ensure national representativeness [4, 22]. On a positive note, the prevalence of quitting intentions witnessed a substantial increase in 2021 compared to 2011, indicating a growing number of individuals contemplating and undertaking smoking cessation efforts, and smoking prevalence has decreased among older adults. The declining trend in current smoker prevalence among adults aged 15 years and older is consistent with findings from the National Health Survey, which reported a decrease from 34.7% in 2010 to 29.7% in 2023 [4, 23].

These findings underscore the challenge of tobacco control efforts in addressing smoking behaviour in Indonesia that are strongly related to complex sociocultural factors. In Indonesia, the complex social dynamics of smoking manifests in many forms as explained by Nichter et al. [24]. Cigarette smoking serves various purposes, including symbolic utility (identity construction, masculinity, modernity, success and control); social utility, which reflects social interaction; regulation utility, which depicts stress‐coping mechanisms and pleasure; and physical regulation utility, which encompasses body rhythms (sleep and wake patterns and appetites). Among teenage boys, cigarette smoking also serves as a symbol of becoming a man because it illustrates maturity and masculinity. Misconceptions about the harm of smoking, peer pressure and parental smoking also play significant roles in teenage smoking behaviour [25, 26]. Strong associations between exposure to second‐hand tobacco smoke and current smoking behaviour in our study provide further evidence regarding the role of home environments. Conversely, there is a significant cultural stigma and disapproval against female smokers which is labelled misbehaviour or having improper attitude despite the growing trend of secular emancipation [25, 27]. Therefore, cigarette smoking in Indonesia translates into a multifaceted cultural and social construct that goes beyond ‘smoking as a commodity’. Consequently, tobacco companies perceive this complex cultural interplay as a strategic way to boost cigarette market sales using intriguing messages related to tradition, modernity, masculinity, politics and nationalism that reflect Indonesians’ ideals [8].

In a country in which smoking has become a social norm, it is interesting to note that more people are trying to stop smoking, whereas smoking prevalence remains high. Prior study suggests various motives behind smoking cessation, ranging from health reasons to social factors [28]. In terms of health reasons, smokers who perceive the negative effects of smoking had higher odds of quitting intention [29]. Meanwhile, social factors comprise a unique perspective that smokers may have concerns about harming social interaction when they try to stop smoking. However, they may also be stigmatised for being smoking parents because it is socially constructed that parents must be an ideal role model for their children [30, 31]. This gap between intention to quit and smoking prevalence may also reveal limitations in support programmes available for those attempting to cease smoking.

This study also reveals that higher exposure to pro‐smoking messages and advertising was not associated with current smoking among Indonesian adults aged 15 years and over after adjusting for sex, perhaps underscoring gender patterns and marketing strategies that mainly target men. This finding differs from prior research in Indonesia among youth participants outlining that exposure to tobacco advertisements, promotions and sponsorships increased the odds of current smoking among Indonesian youths aged 13–15 years [11]. Another survey in Indonesia among 2115 high school students also highlights that exposure to cigarette advertisements increased the odds of smoking initiation and current smoking [32]. However, this study is limited in its inclusion of minors and does not fully represent the general Indonesian population. Moreover, tobacco industries have long been targeting advertisements specifically among children and adolescents, including promoting cigarettes at the point of sale located near schools [31]. There are four common strategies identified to attract young smokers: displaying cigarettes along with snacks and sugary drinks, displaying cigarette posters within youths’ views, promoting flavoured cigarettes and selling single‐stick cigarettes [33]. Moreover, for these traditional avenues for tobacco promotion to young people in Indonesia, there is increasing evidence that new advertising and promotion channels, which are less regulated, particularly on social media, may help explain the increased prevalence of youth smokers in 2021 [34].

It is noteworthy that among males, exposure to pro‐smoking messages is not associated with current smoking behaviour but is associated with increased likelihood of quitting smoking attempts in the last 12 months. This study's finding aligns with prior studies among Indonesian youths that exposure to cigarette advertisements increased the odds of contemplating and action phases of quitting smoking [13]. In this study, exposure to pro‐smoking media was significantly associated with exposure to anti‐smoking media (see Supporting Information 4), indicating that individuals frequently encounter both types of messages concurrently. This overlap may partly explain the observed positive association between pro‐smoking media exposure and quit attempts. Individuals exposed to both pro‐ and anti‐smoking messages may experience conflicting cues, which could trigger cognitive dissonance or reflection on their smoking behaviour, ultimately motivating attempts to quit. Thus, the observed relationship between pro‐smoking media and cessation behaviour may not be purely causal but influenced by concurrent exposure to anti‐smoking messages.

In Indonesia, tobacco advertising and packaging must contain anti‐smoking messages. Government Regulation Number 109 of 2012 mandates health warnings in the form of both images and text to be included as a minimum of 10% of the total duration or 15% of the total area of the advertisement [35]. This regulation also prohibits the physical display of cigarettes, using persuasive and misleading messages in advertisements, using explicit product names such as ‘cigarettes’, and displaying children, adolescents, pregnant women or cartoon characters [35]. According to the Transtheoretical Model of Behavioural Change, this complex interaction of pro‐smoking messages containing anti‐smoking messages can influence individuals’ contemplation and preparation stages by creating cognitive dissonance, leading them to reassess their smoking habits [36, 37]. However, smoking cessation intention at the individual level translates into a more complex interplay between internal and external motivation [38].

Exposure to anti‐smoking messages is a protective factor against current smoking and is associated with quitting intention. Prior studies revealed consistent findings in terms of current smoking behaviour and quitting intention [39, 40, 41, 42, 43, 44, 45]. In contrast, a previous study in Indonesia suggests that adolescents who were exposed to anti‐smoking media have higher odds of being current smokers [18]. These inconsistent findings may be due to campaign coverage, exposure level and duration, and type of messages which significantly affect programmes’ efficacy [41].

The stratification by sex in this study primarily focuses on male smokers due to the limited sample size of female smokers. Given the importance of examining gender‐specific effects, future research with a larger sample size of female smokers is crucial for a deeper understanding of smoking behaviour and quitting intention dynamics among women. Additionally, this study is unable to thoroughly explore the attitudes and motivations driving individuals to continue smoking and their efforts to quit in relation to different media exposures. Therefore, future research could employ qualitative or mixed method designs to address these aspects. Furthermore, as this study's analysis uses weighting with an estimated sample size, it may affect the precision of the estimated odds ratios. For the association between exposure to pro‐smoking messages and attempts to quit in the last 12 months, the point estimates (95% CI) range from 1.01 to 1.64, indicating a weak association. Consequently, future studies should use larger sample sizes and ensure greater sample variability to improve precision and statistical power. Moreover, as this study employs a repeated cross‐sectional design, we are unable to examine longitudinal changes among individuals that establish causality. However, these surveys are the best nationally representative data sources available that provide a valuable insight into diverse aspects of media exposure. Moreover, as this study employs a repeated cross‐sectional design, we are unable to examine longitudinal changes among individuals that establish causality.

## 5 Conclusion

Despite a slight decrease in smoking prevalence from 2011 to 2021, male smoking prevalence remains high, and more adolescents aged 15–24 are becoming current smokers. This study also highlights the significant role of anti‐smoking messaging in its association with lower likelihood of current smoking as well as increased likelihood of quitting intention and attempts to quit, whereas pro‐smoking messaging was not associated with increased smoking likelihood. Therefore, the current anti‐smoking campaign in Indonesia has the potential to improve individuals’ quitting intentions. This provides the Indonesian government with strong evidence of the value of continuing and strengthening regulation of tobacco advertisements, improving public awareness through anti‐smoking campaigns which are specifically aimed at male audiences, and supporting smoking cessation programmes.

## Author Contributions

SF, MK, INS PASA conceptualized the research led by SF. SF obtained resources (data access), curated the data and conducted first formal analysis. SF and MK prepared first draft, SF, MK, INS, PASA reviewed and edited drafts and revision versions of manuscript. MK carried out project administration and supervision.

## Funding

The authors have nothing to report.

## Ethics Statement

This study utilised secondary, de‐identified data from the WHO Global Adults Tobacco Survey data source database. As such, no ethical approval was required.

## Conflicts of Interest

The authors declare no conflicts of interest.

## Supporting information




**Supporting Information 1** Description of study variables, original questions and measurements.
**Supporting Information 2** Prevalence of current smoking behaviour in 2021 (*N* = 9156) and 2011 (*N* = 8305) in weighted row percentage.
**Supporting Information 3** Prevalence of attempts to quit in the last 12 months in 2011 (*N* = 2853) and 2021 (*N* = 2874) in weighted row percentage.
**Supporting Information 4** Prevalence of thoughts of quitting in 2011 (*N* = 2853) and 2021 (*N *= 2874) in weighted row percentage.
**Supporting Information 5** Socio‐demographic and exposure characteristics of study sample and smoking behaviour among Indonesian adults in 2021 (*N *= 9156) in weighted row percentage.
**Supporting Information 6** Description of the Global Adult Tobacco Survey Questionnaire.
**Supporting Information 7** Stata Commands.

## Data Availability

The data used in this study are from the WHO Global Adults Tobacco Survey data source. Data are available to the public upon request with data access available here https://extranet.who.int/ncdsmicrodata/index.php/catalog/955.
