# Supplementary material for: Smoking Patterns, Quitting Intentions and Associations With Pro‐ and Anti‐Smoking Messages: Insights From the Indonesian GATS Survey (2011 and 2021)
Source: Public Health Chall. 2026 Feb 23;5(1):e70202. doi: 10.1002/puh2.70202 (PMC12928039; doi:10.1002/puh2.70202)
Supplement: Supplementary file 1 — Supporting Information 1 Description of study variables, original questions and measurements. Supporting Information 2 Prevalence of current smoking behaviour in 2021 (N = 9156) and 2011 (N = 8305) in weighted row percentage. Supporting Information 3 Prevalence of attempts to quit in the last 12 months in 2011 (N = 2853) and 2021 (N = 2874) in weighted row percentage. Supporting Information 4 Prevalence of thoughts of quitting in 2011 (N = 2853) and 2021 (N = 2874) in weighted row percentage. Supporting Information 5 Socio‐demographic and exposure characteristics of study sample and smoking behaviour among Indonesian adults in 2021 (N = 9156) in weighted row percentage. Supporting Information 6 Description of the Global Adult Tobacco Survey Questionnaire. Supporting Information 7 Stata Commands. [file PUH2-5-e70202-s001.docx]

**Supplementary Materials**

The following pages contain the detailed description of survey variables and instruments used in the study to collect data on participants' demographics, smoking behaviour, exposure to smoking messages, and intentions to quit smoking. The survey instrument is organised into sections for clarity and ease of reference.

Please refer to the following list to quickly locate specific sections:

Supplementary 1. Description of Study Variables, original questions, and measurements...............................................................................................................(Page 2)

Supplementary 2. Prevalence of current smoking behaviour in 2021 (N=9156) and 2011 (N=8305) in weighted row percentage in weighted row percentage………………………………………………………………………...…………. (Page 7)

Supplementary 3. Prevalence of attempts to quit in the last 12 months in 2011 (N= 2853) and 2021 (N=2874) in weighted row percentage………………………………………………………………………………....…(Page 9)

Supplementary 4. Prevalence of thoughts of quitting in 2011 (N= 2853) and 2021 (N=2874) in weighted row percentage…………………………………………………………………. (Page 11)

Supplementary 5. Socio-demographic and exposure characteristics of study sample and smoking behaviour among Indonesian adults in 2021 (N=9156) in weighted row percentage.……………………..………………………………………………………… (Page 13)

Supplementary 6. Description of GATS Questionnaire

…………... ….……………………………………………………………………………….(Page 15)

Supplementary 7.Stata Commands…...…………………………………………..…….. (Page 16)

**Supplementary 1. Description of Study Variables, original questions, and measurements.**

Outcome variables

1. Smoking Behaviour

Smoking behaviour is a binary data that is classified into current smokers (coded as 1) and current non-smokers coded as 0)

Original question:

“The following questions are about the use of different types of tobacco products. There are four categories of products that I will be asking you about separately: “classic” tobacco smoking products; electronic cigarettes such as SMOK, Ploom, Blu; heated tobacco products such as IQOS, Glo; and smokeless tobacco.

I would now like to ask you some questions about smoking tobacco, including white cigarettes, kretek cigarettes, cigars, pipes, shisha with tobacco. This includes all products where you burn the tobacco as you smoke it. Do you currently smoke tobacco on a daily basis, less than daily, or not at all?”

Measurement:

Respondent who answered “daily and less than daily” is categorised as current smokers (coded as 1), while those who answered “not at all” is categorised as non-current smokers (is coded as 0).

1. Quitting Intention Behaviour
   1. Attempts to quit in the last 12 months.

Attempts to quit in the last 12 months is a binary data that is classified into trying to quit (coded as 1) and not trying to quit (coded as 0).

Original question:

“The next questions ask about any attempts to stop smoking that you might have made during the past 12 months. Please think about tobacco smoking: During the past 12 months, have you tried to stop smoking?”

Measurement:

Respondents who answered “yes” is categorised as “trying to quit” (is coded as 1), while those who answered “no” is categorised as “not trying to quit” (is coded as 0). 3 refused responses were classified as missing data.

- 1. Thoughts of quitting (Yes and No)

Thoughts of quitting in the last 12 months is a binary data that is classified into yes (coded as 1) and no (coded as 0).

Original question:

“Which of the following best describes your thinking about quitting smoking? I am planning to quit within the next month, I am thinking about quitting within the next 12 months, I will quit someday but not within the next 12 months, or I am not interested in quitting?”

Measurement:

Respondents who answered “quit within the next month”, “thinking within the next 12 months”, “quit someday but not in the next 12 months” are categorised as “think to quit” (is coded as 1) while those who answered “not interested in quitting” and “don’t know” are categorised as “not thinking to quit” (is coded as 0). 7 refused responses were classified as missing data.

Exposure variables

1. Antismoking messages

Antismoking messages is a categorical data that is classified into exposed (coded as 1) and not exposed (coded as 2)

Original question

“In the last 30 days, have you noticed information about the dangers of smoking white or kretek cigarettes or that encourages quitting in any of the following places? in newspapers or in magazines? on television? on the radio? on billboards? On the internet? somewhere else?”

Measurement:

6 questions were identified regarding the source of antismoking messages noticed by the respondents in which 1 score is applied in every “yes” answer. Then, total score is calculated for each respondent. Following this, mean score is calculated for all respondents (mean total=1.2 rounded to 1). Therefore, respondent who have total score ≥ 1 is classified as “exposed” and respondent who have 0 total score is classified as “not exposed”.

1. Pro smoking messages

Antismoking messages is a categorical data that is classified into higher (coded as 1) and lower (coded as 2)

Original question:

“I will now ask you about noticing marketing of tobacco products. I will ask you separately about noticing marketing of traditional cigarettes including white and kretek cigarettes and noticing marketing of electronic cigarettes such as SMOK, Ploom, Blu.”

“In the last 30 days, have you noticed any advertisements or signs promoting white or kretek cigarettes in the following places? In stores where cigarettes are sold? On television? On the radio? On billboards? On posters? In newspapers or magazines? In cinemas? On the internet? On public transportation vehicles or stations? On public walls? Anywhere else?”

“In the last 30 days, have you noticed: any sport or sporting event that is associated with white or kretek cigarette brands or companies?

“In the last 30 days, have you noticed any music, theatre, art, or fashion events that are associated with white or kretek cigarette brands or companies?”

“In the last 30 days, have you noticed: any of the following types of white or kretek cigarette promotions? Free samples of cigarettes? Cigarettes at sale prices? Coupons for cigarettes? Free gifts or special discount offers? Clothing or other items with a cigarette brand name or logo? Cigarette promotions in the mail?”

Measurement:

18 questions were identified regarding the source of pro-smoking messages noticed by the respondents in which 1 score is applied in every “yes” answer. Then, total score is calculated for each respondent. Following this, mean score is calculated for all respondents (mean score = 2.5 rounded to 3). Respondent who have total score >3 (4 or more type of media exposure) is classified as “higher exposure” (is coded as 1) and respondents who have total score ≤3 (3 and less type of media exposure) is classified as “lower” (is coded as 2).

1. Age group

Original Question:

“What is the year of your date of birth? What is the month of your date of birth?”

Measurement:

In the original dataset, age variable is available as continuous and is classified into 4 categories: 15-18 (is coded as 1), 19-30 (is coded as 2), 31-50 (is coded as 3), and >51 years old (is coded as 4).

1. Sex

Original question:

“[record gender from observation. Ask if necessary.]”

Measurement:

In the dataset, sex is classified into male and female in which male is coded as 1 and female is coded as 2.

1. Education Level

Original question:

“What is the highest level of education you have completed? 1) less than primary school completed; 2) primary school completed; 3) secondary school completed; 4) high school completed; 5) college/university completed; 6) post graduate degree completed; 7) Don’t know; 8) Refused.”

Measurement:

Less than primary school and primary school is categorised as “primary school and under” (is coded as 1). Secondary school completed is coded as 2. High school completed is coded as 3. College/university and post graduate degree completed is coded as 4.

1 “refused” and 2 “don’t know” answer are treated as missing and are automatically omitted in regression analysis.

1. Residence

Measurement

In the dataset, residence is classified into urban and rural in which urban is coded as 1 and rural is coded as 2.

1. Work Status

Original question:

“Which of the following best describes your main work status over the past 12 months? Government employee, non-government employee, self-employed, student, homemaker, retired, unemployed-able to work, or unemployed-unable to work? [include subsistence farming as self-employed]”

Measurement:

Government employee and non-government employee are classified as “employed” (is coded as 1). “Self-employed” is coded as 2. Student, homemaker, and retired are classified as “non-labour force” (is coded as 3). Unemployed, able to work and unemployed unable to work are classified as “unemployed” (is coded as 4). 3 “don’t know” responses are treated as missing and are automatically omitted in regression analysis.

1. Second-hand smoke
2. Second-hand smoking exposure at home:

“Which of the following best describes the rules about smoking inside of your home: Smoking is allowed inside of your home, smoking is generally not allowed inside of your home but there are exceptions, smoking is never allowed inside of your home, or there are no rules about smoking in your home?”

“How often does anyone smoke inside your home? Would you say daily, weekly, monthly, less than monthly, or never?”

Measurement: 1 score is applied to respondent who answered, “Smoking is allowed inside of your home, smoking is generally not allowed inside of your home but there are exceptions”. 0 score is applied to respondent who answered, “smoking is never allowed”. For those who answered, “no rules”, I refer to the next question “how often does anyone smoke inside your home”. 1 score is applied for those who answered “daily, weekly, monthly, less than monthly”. 0 score is applied for those who answered “never”. Then, new variable for second-hand smoking exposure at home is created (1 =yes/exposed, 0=no/not exposed).

1. Second-hand smoking exposure in public places:

“During the past 30 days, did anyone smoke in: indoor areas where you work?”

“Did anyone smoke inside of any government buildings or government offices that you visited in the past 30 days?”

“Did anyone smoke inside of any health care facilities that you visited in the past 30 days?”

“Did anyone smoke inside of any restaurants that you visited in the past 30 days?”

“Did anyone smoke inside of any cafes or coffee shops that you visited in the past 30 days?”

“Did anyone smoke inside of any public transportation that you used in the past 30 days?”

“Did anyone smoke inside of any university buildings that you visited in the past 30 days?”

“Did anyone smoke inside of any school or educational facility buildings that you visited in the past 30 days?”

“Did anyone smoke inside of any religious facilities that you visited in the past 30 days?”

“During the past 30 days, did anyone smoke inside any other public places while you were visiting?”

Measurement: 1 score is applied to every “yes” answer.

Then, new variable of “second-hand smoke” is created by summing up variable second-hand smoking at home and second-hand smoking in public places. Following this, total mean score is calculated (mean=2.1 rounded to 2). Those who scored >2 (3 or more) second-hand smoking exposure are classified as “higher exposure” and those who scored ≤ 2 (2 and less) exposure are classified as “lower exposure”.

1. Knowledge about smoking harm

Original question:

“Based on what you know or believe: does smoking tobacco cause serious illness?”

“Based on what you know or believe, does smoking tobacco cause the following: stroke?”

“Based on what you know or believe, does smoking tobacco cause the following: heart attack?”

“Based on what you know or believe, does smoking tobacco cause the following: lung cancer?”

“Based on what you know or believe, does smoking tobacco cause the following: COPD (Chronic Obstructive Pulmonary Disease?”

“Based on what you know or believe, does smoking tobacco cause the following: bladder cancer?”

“Based on what you know or believe, does smoking tobacco cause the following: stomach cancer?”

“Based on what you know or believe, does smoking tobacco cause the following: oral cancer?”

“Based on what you know or believe, does smoking tobacco cause the following: cervix cancer?”

“Based on what you know or believe, does smoking tobacco cause the following: breast cancer?”

“Based on what you know or believe, does smoking tobacco cause the following: larynx cancer?”

“Based on what you know or believe, does smoking tobacco cause the following: premature birth?”

“Based on what you know or believe, does smoking tobacco cause the following: bone loss?”

Measurement:

1 score is applied to every yes answer and then total individual score is calculated. Following this, mean score is calculated for all respondents (mean score = 7.1 rounded to 7). Respondents who have total score of ≥7 is categorised as having “higher” knowledge about the harm of smoking. While respondent who have total score of ≤6 is categorised as “lower” knowledge.

1. Smoking dependency

Original question:

“How soon after you wake up do you usually have your first smoke? Would you say within 5 minutes, 6 to 30 minutes, 31 to 60 minutes, or more than 60 minutes?”

Measurement

Respondents who answered within 5 minutes, 6 to 30 minutes, 31 to 60 minutes are categorise as “higher dependency” (coded as 1) and respondents who answered more than 60 minutes are categorised as “lower dependency” (coded as 2).

1. Smoking level

Original question:

“How many of the following do you currently smoke in a day: Manufactured white cigarettes?”

“How many of the following do you currently smoke in a day: Hand-rolled (RYO) cigarettes?”

“How many of the following do you currently smoke in a day: Kretek cigarettes?”

“How many of the following do you currently smoke in a day: Pipes full of tobacco?”

“How many of the following do you currently smoke in a day: Cigars?”

“How many of the following do you currently smoke in a day: shisha sessions?”

Measurement:

The number of cigarettes consumed per day is discrete data. Firstly, total cigarettes consumed per day by each individual is calculated. Then, the total mean of all respondents is calculated (mean=13). Respondents who consumed >13 (14 or more) cigarettes per day is categorised as “heavy smokers” (is coded as 1) and individuals who consumed ≤ 13 cigarettes per day is categorised as “light smokers” (is coded as 2).

**Supplementary 2. Prevalence of current smoking behaviour in 2021 (N=9156) and 2011 (N=8305) in weighted row percentage**.

| **Variable** | **GATS 2011** | | | | | **GATS 2021** | | | | | **Changes of quitting behaviour** |
| --- | --- | --- | --- | --- | --- | --- | --- | --- | --- | --- | --- |
|  | **Current Smokers** | | **Current Non-smokers** | | **p-value** | **Current Smokers** | | **Current Non-smokers** | | **p-value** |  |
|  | **n** | **%** | **n** | **%** |  | **n** | **%** | **n** | **%** |  | **p-value** |
| **Age** |  |  |  |  |  |  |  |  |  |  |  |
| 15 - 18 | 101 | 17.45 | 490 | 82.55 | < 0.01 | 156 | 23.64 | 484 | 76.36 | < 0.01 | < 0.01 |
| 19 - 30 | 624 | 34.73 | 1324 | 65.27 |  | 516 | 35.07 | 1061 | 64.93 |  | < 0.01 |
| 31 - 50 | 1384 | 38.67 | 2250 | 61.33 |  | 1240 | 36.19 | 2487 | 63.81 |  | < 0.01 |
| > 51 | 746 | 36.07 | 1386 | 63.92 |  | 965 | 31.28 | 2247 | 68.72 |  | < 0.01 |
|  |  |  |  |  |  |  |  |  |  |  |  |
| **Sex** |  |  |  |  |  |  |  |  |  |  |  |
| Male | 2720 | 67.04 | 1228 | 32.96 | < 0.01 | 2776 | 64.71 | 1536 | 35.29 | < 0.01 | < 0.01 |
| Female | 135 | 2.66 | 4222 | 97.34 |  | 101 | 2.25 | 4743 | 97.75 |  | < 0.01 |
|  |  |  |  |  |  |  |  |  |  |  |  |
| **Education Level** |  |  |  |  |  |  |  |  |  |  |  |
| Primary school and under | 1612 | 37.54 | 2807 | 62.46 | < 0.01 | 1340 | 34.9 | 2828 | 65.1 | < 0.01 | < 0.01 |
| Secondary school | 486 | 31.9 | 1039 | 68.1 |  | 570 | 33.49 | 1169 | 66.51 |  | < 0.01 |
| High School | 607 | 33.74 | 1178 | 66.25 |  | 797 | 35.48 | 1614 | 64.52 |  | < 0.01 |
| College and postgrad | 150 | 27.64 | 423 | 72.36 |  | 170 | 22.21 | 665 | 77.79 |  | < 0.01 |
|  |  |  |  |  |  |  |  |  |  |  |  |
| **Residence** |  |  |  |  |  |  |  |  |  |  |  |
| Urban | 1307 | 31.95 | 2795 | 68.05 | < 0.01 | 1221 | 32.16 | 2870 | 67.84 | < 0.01 | < 0.01 |
| Rural | 1548 | 37.66 | 2655 | 62.34 |  | 1656 | 35.22 | 3409 | 64.78 |  | < 0.01 |
|  |  |  |  |  |  |  |  |  |  |  |  |
| **Work Status** |  |  |  |  |  |  |  |  |  |  |  |
| Employed | 1080 | 46.76 | 1261 | 53.24 | < 0.01 | 840 | 45.33 | 1138 | 54.67 | < 0.01 | < 0.01 |
| Self-Employed | 1454 | 50.33 | 1559 | 49.67 |  | 1545 | 49.11 | 1829 | 50.89 |  | < 0.01 |
| Non-Labour force | 144 | 6.57 | 2262 | 93.43 |  | 231 | 8.18 | 2909 | 91.82 |  | < 0.01 |
| Unemployed | 175 | 31.73 | 367 | 68.27 |  | 261 | 43.23 | 400 | 56.77 |  | < 0.01 |
|  |  |  |  |  |  |  |  |  |  |  |  |
| **Second-hand smoke** |  |  |  |  |  |  |  |  |  |  |  |
| Higher | 1063 | 41.33 | 1511 | 58.67 | < 0.01 | 1318 | 47.63 | 1545 | 52.37 | < 0.01 | < 0.01 |
| Lower | 1792 | 31.63 | 3939 | 68.37 |  | 1559 | 25.83 | 4734 | 74.17 |  | < 0.01 |
|  |  |  |  |  |  |  |  |  |  |  |  |
| **Knowledge about smoking harm** |  |  |  |  |  |  |  |  |  |  |  |
| Higher | 906 | 29.21 | 2198 | 70.79 | < 0.01 | 1450 | 28.97 | 3873 | 71.03 | < 0.01 | < 0.01 |
| Lower | 1949 | 38.33 | 3252 | 61.67 |  | 1427 | 40.75 | 2406 | 59.25 |  | < 0.01 |
|  |  |  |  |  |  |  |  |  |  |  |  |
| **Among current smokers only (column percentage)** |  |  |  |  |  |  |  |  |  |  |  |
| **Attempts to stop smoking in the last 12 months** |  |  |  |  |  |  |  |  |  |  |  |
| yes | 821 | 29.11^‡^ |  |  |  | 1158 | 42.7^‡^ |  |  |  |  |
| no | 2032 | 70.89^‡^ |  |  |  | 1716 | 57.3^‡^ |  |  |  |  |
|  |  |  |  |  |  |  |  |  |  |  |  |
| **Thoughts of Quitting** |  |  |  |  |  |  |  |  |  |  |  |
| Yes | 1408 | 48.72^‡^ |  |  |  | 1738 | 63.32^‡^ |  |  |  | < 0.01 |
| No | 1447 | 51.28^‡^ |  |  |  | 1139 | 36.68^‡^ |  |  |  | < 0.01 |
|  |  |  |  |  |  |  |  |  |  |  |  |
| **Among daily smokers** |  |  |  |  |  |  |  |  |  |  |  |
| smoking dependency |  |  |  |  |  |  |  |  |  |  |  |
| higher | 1,653 | 67.71^‡^ |  |  |  | 1,374 | 59.06^‡^ |  |  |  | < 0.01 |
| lower | 769 | 32.29^‡^ |  |  |  | 956 | 40.94^‡^ |  |  |  | < 0.01 |
|  |  |  |  |  |  |  |  |  |  |  |  |
| **Smoking level** |  |  |  |  |  |  |  |  |  |  |  |
| Heavy | 1,518 | 62.33^‡^ |  |  |  | 811 | 33.56^‡^ |  |  |  | < 0.01 |
| Light | 907 | 37.67^‡^ |  |  |  | 1,521 | 66.44^‡^ |  |  |  | < 0.01 |
| ^‡^ Column percentage | | | | | | | | | | | |

**Supplementary 3. Prevalence of attempts to quit in the last 12 months in 2011 (N= 2853) and 2021 (N=2874) in weighted row percentage**

| **Variable** | **GATS 2011** | | | | | **GATS 2021** | | | | | **Changes of quitting behaviour** |
| --- | --- | --- | --- | --- | --- | --- | --- | --- | --- | --- | --- |
|  | **Trying to quit**  **(Yes)** | | **Not Trying to quit (No)** | | **p-value** | **Trying to quit**  **(Yes)** | | **Not trying to quit**  **(No)** | | **p-value** |  |
|  | **n** | **%** | **n** | **%** |  | **n** | **%** | **n** | **%** |  | **p-value** |
| **Age** |  |  |  |  |  |  |  |  |  |  |  |
| 15 - 18 | 38 | 36.57 | 63 | 63.43 | 0.07 | 87 | 56.98 | 69 | 43.02 | < 0.01 | < 0.01 |
| 19 - 30 | 200 | 31.45 | 424 | 68.55 |  | 244 | 47.9 | 272 | 5.21 |  | < 0.01 |
| 31 - 50 | 403 | 28.55 | 980 | 71.45 |  | 469 | 39.39 | 769 | 60.61 |  | < 0.01 |
| > 51 | 180 | 25.54 | 565 | 74.46 |  | 358 | 40.06 | 606 | 59.94 |  | < 0.01 |
|  |  |  |  |  |  |  |  |  |  |  |  |
| **Sex** |  |  |  |  |  |  |  |  |  |  |  |
| Male | 772 | 28.66 | 1,947 | 71.34 | <0.01 | 1,112 | 42.62 | 1,662 | 57.38 | 0.73 | < 0.01 |
| Female | 49 | 40.57 | 85 | 59.43 |  | 46 | 44.98 | 54 | 55.02 |  | < 0.01 |
|  |  |  |  |  |  |  |  |  |  |  |  |
| **Education Level** |  |  |  |  |  |  |  |  |  |  |  |
| Primary school and under | 387 | 24.64 | 1,224 | 75.36 | < 0.01 | 463 | 36.96 | 874 | 63.04 | < 0.01 | < 0.01 |
| Secondary school | 159 | 31.75 | 327 | 68.25 |  | 242 | 43.97 | 328 | 56.03 |  | < 0.01 |
| High School | 218 | 35.53 | 389 | 64.47 |  | 371 | 47.95 | 426 | 52.05 |  | < 0.01 |
| College and postgrad | 57 | 37.18 | 92 | 62.82 |  | 82 | 47.73 | 88 | 52.27 |  | < 0.01 |
|  |  |  |  |  |  |  |  |  |  |  |  |
| **Residence** |  |  |  |  |  |  |  |  |  |  |  |
| Urban | 456 | 34.22 | 850 | 65.78 | 0.02 | 553 | 47.07 | 668 | 52.93 | <0.01 | < 0.01 |
| Rural | 365 | 24.74 | 1,182 | 75.26 |  | 605 | 37.32 | 1,048 | 62.68 |  | < 0.01 |
|  |  |  |  |  |  |  |  |  |  |  |  |
| **Work Status** |  |  |  |  |  |  |  |  |  |  |  |
| Employed | 359 | 33.58 | 721 | 66.42 | < 0.01 | 353 | 43.98 | 487 | 56.02 | < 0.01 | < 0.01 |
| Self-Employed | 363 | 25.3 | 1,091 | 74.7 |  | 551 | 38.14 | 992 | 61.86 |  | < 0.01 |
| Non-Labour force | 59 | 38.03 | 85 | 61.97 |  | 125 | 53.67 | 105 | 46.33 |  | < 0.01 |
| Unemployed | 38 | 23.05 | 135 | 76.95 |  | 129 | 52.02 | 132 | 47.98 |  | < 0.01 |
|  |  |  |  |  |  |  |  |  |  |  |  |
| **Second-hand smoke** |  |  |  |  |  |  |  |  |  |  |  |
| Higher | 355 | 33.27 | 708 | 66.73 | < 0.01 | 560 | 44.94 | 758 | 55.06 | 0.08 | < 0.01 |
| Lower | 466 | 26.47 | 1,324 | 73.53 |  | 598 | 40.47 | 958 | 59.53 |  | < 0.01 |
|  |  |  |  |  |  |  |  |  |  |  |  |
| **Knowledge about smoking harm** |  |  |  |  |  |  |  |  |  |  |  |
| Higher | 360 | 40.83 | 545 | 59.17 | < 0.01 | 679 | 49.2 | 769 | 50.8 | < 0.01 | < 0.01 |
| Lower | 461 | 23.45 | 1,487 | 76.55 |  | 479 | 35.23 | 947 | 64.77 |  | < 0.01 |
|  |  |  |  |  |  |  |  |  |  |  |  |
| **Thoughts of Quitting** |  |  |  |  |  |  |  |  |  |  |  |
| Yes | 580 | 41.61 | 828 | 58.39 | < 0.01 | 891 | 53.11 | 845 | 46.89 | < 0.01 | < 0.01 |
| No | 241 | 17.2 | 1,204 | 82.8 |  | 267 | 24.73 | 871 | 75.27 |  | < 0.01 |
|  |  |  |  |  |  |  |  |  |  |  |  |
| **Among daily smokers** |  |  |  |  |  |  |  |  |  |  |  |
| smoking dependency |  |  |  |  |  |  |  |  |  |  |  |
| higher | 390 | 24.27 | 1,263 | 75.73 | 0.04 | 437 | 33.49 | 937 | 66.51 | < 0.01 | < 0.01 |
| lower | 240 | 31.1 | 528 | 68.9 |  | 395 | 44.23 | 560 | 55.77 |  | < 0.01 |
|  |  |  |  |  |  |  |  |  |  |  |  |
| **Smoking level** |  |  |  |  |  |  |  |  |  |  |  |
| Heavy | 352 | 23.71 | 1,166 | 76.29 | < 0.01 | 265 | 34.85 | 546 | 65.15 | 0.06 | < 0.01 |
| Light | 278 | 30.95 | 628 | 69.05 |  | 568 | 39.44 | 952 | 60.56 |  | < 0.01 |

**Suplementary 4. Prevalence of thoughts of quitting in 2011 (N= 2853) and 2021 (N=2874) in weighted row percentage**

|  | **GATS 2011** | | | | | **GATS 2021** | | | | |  |
| --- | --- | --- | --- | --- | --- | --- | --- | --- | --- | --- | --- |
| **Variable** | **Thoughts of quitting** | | | | | **Thoughts of quitting** | | | | | **Changes of quitting behaviour** |
|  | **Yes** | | **No** | | **p-value** | **Yes** | | **No** | | **p-value** |  |
|  | **n** | **%** | **n** | **%** |  | **n** | **%** | **n** | **%** |  | **p-value** |
| **Age** |  |  |  |  |  |  |  |  |  |  |  |
| 15 - 18 | 52 | 53.43 | 49 | 46.57 | < 0.01 | 102 | 63.69 | 54 | 36.31 | < 0.01 | < 0.01 |
| 19 - 30 | 319 | 49.76 | 304 | 50.24 |  | 346 | 67.21 | 170 | 32.79 |  | < 0.01 |
| 31 - 50 | 729 | 51.36 | 652 | 48.64 |  | 778 | 64.82 | 462 | 35.18 |  | < 0.01 |
| > 51 | 308 | 41.01 | 438 | 58.99 |  | 512 | 56.94 | 453 | 43.06 |  | < 0.01 |
|  |  |  |  |  |  |  |  |  |  |  |  |
| **Sex** |  |  |  |  |  |  |  |  |  |  |  |
| Male | 1,343 | 48.92 | 1,373 | 51.08 | 0.64 | 1,674 | 63.32 | 1,102 | 36.68 | 0.98 | < 0.01 |
| Female | 65 | 45.78 | 70 | 54.22 |  | 64 | 63.19 | 37 | 36.81 |  | < 0.01 |
|  |  |  |  |  |  |  |  |  |  |  |  |
| **Education Level** |  |  |  |  |  |  |  |  |  |  |  |
| Primary school and under | 668 | 40.81 | 944 | 59.19 | < 0.01 | 688 | 53.44 | 652 | 46.56 | < 0.01 | < 0.01 |
| Secondary school | 254 | 51.41 | 232 | 48.59 |  | 357 | 63.94 | 213 | 36.06 |  | < 0.01 |
| High School | 380 | 61.33 | 223 | 38.67 |  | 562 | 71.87 | 235 | 28.13 |  | < 0.01 |
| College and postgrad | 106 | 66.42 | 44 | 33.58 |  | 131 | 79.01 | 39 | 20.99 |  | < 0.01 |
|  |  |  |  |  |  |  |  |  |  |  |  |
| **Residence** |  |  |  |  |  |  |  |  |  |  |  |
| Urban | 682 | 50.64 | 622 | 49.36 | 0.56 | 820 | 69.4 | 401 | 30.6 | < 0.01 | < 0.01 |
| Rural | 726 | 47.22 | 821 | 52.78 |  | 918 | 55.85 | 738 | 44.15 |  | < 0.01 |
|  |  |  |  |  |  |  |  |  |  |  |  |
| **Work Status** |  |  |  |  |  |  |  |  |  |  |  |
| Employed | 567 | 50.62 | 512 | 49.38 | 0.31 | 573 | 69.76 | 267 | 30.24 | < 0.01 | < 0.01 |
| Self-Employed | 689 | 47.64 | 762 | 52.36 |  | 864 | 59.07 | 681 | 40.93 |  | < 0.01 |
| Non-Labour force | 84 | 54.67 | 60 | 45.33 |  | 153 | 66.02 | 78 | 33.98 |  | < 0.01 |
| Unemployed | 66 | 41.06 | 109 | 58.94 |  | 148 | 60.99 | 113 | 39.01 |  | < 0.01 |
|  |  |  |  |  |  |  |  |  |  |  |  |
| **Second-hand smoke** |  |  |  |  |  |  |  |  |  |  |  |
| Higher | 582 | 53 | 479 | 47 | 0.03 | 855 | 66.96 | 463 | 33.04 | < 0.01 | < 0.01 |
| Lower | 826 | 46.14 | 964 | 53.86 |  | 883 | 59.7 | 676 | 40.3 |  |  |
|  |  |  |  |  |  |  |  |  |  |  |  |
| **Knowledge about smoking harm** |  |  |  |  |  |  |  |  |  |  |  |
| Higher | 576 | 61.7 | 327 | 38.3 | < 0.01 | 1,061 | 74.96 | 389 | 25.04 | < 0.01 | < 0.01 |
| Lower | 832 | 42.58 | 1,116 | 57.42 |  | 677 | 49.92 | 750 | 50.08 |  |  |
|  |  |  |  |  |  |  |  |  |  |  |  |
| **Trying to stop smoking in the last 12 months** |  |  |  |  |  |  |  |  |  |  |  |
| Yes | 580 | 69.97 | 238 | 30.03 | < 0.01 | 891 | 78.75 | 267 | 21.25 | < 0.01 | < 0.01 |
| No | 828 | 40.2 | 1,203 | 59.8 |  | 845 | 51.8 | 871 | 48.2 |  | < 0.01 |
|  |  |  |  |  |  |  |  |  |  |  |  |
| **Among daily smokers** |  |  |  |  |  |  |  |  |  |  |  |
| **smoking dependency** |  |  |  |  |  |  |  |  |  |  |  |
| higher | 765 | 45.45 | 887 | 54.55 | 0.29 | 700 | 53.85 | 674 | 46.15 | < 0.01 | < 0.01 |
| lower | 384 | 49.89 | 385 | 50.11 |  | 640 | 6.94 | 316 | 30.6 |  | < 0.01 |
|  |  |  |  |  |  |  |  |  |  |  |  |
| **Smoking level** |  |  |  |  |  |  |  |  |  |  |  |
| Heavy | 697 | 54.85 | 819 | 45.15 | 0.14 | 417 | 52.7 | 394 | 47.3 | < 0.01 | < 0.01 |
| Light | 452 | 50.35 | 455 | 49.65 |  | 923 | 63.95 | 598 | 36.05 |  |  |

**Supplementary 5. Socio-demographic and exposure characteristics of study sample and smoking behaviour among Indonesian adults in 2021 (N=9156) in weighted row percentage.**

| **Variable** | **GATS 2021** | | | | | | | |  |
| --- | --- | --- | --- | --- | --- | --- | --- | --- | --- |
|  | **Current Smoker** | | | | **Current Non-Smoker** | | | | **p-value** |
|  | **Daily Smoker** | | **Occasional Smoker** | | **Former Smoker** | | **Never smoker** | |  |
|  | **n** | **%** | **n** | **%** | **n** | **%** | **n** | **%** |  |
| **Age** |  |  |  |  |  |  |  |  |  |
| 15 - 18 | 74 | 11.55 | 82 | 12.1 | 48 | 7.94 | 436 | 68.42 | <0.01 |
| 19 - 30 | 409 | 27.79 | 107 | 7.28 | 117 | 7.54 | 944 | 57.39 |  |
| 31 - 50 | 1,060 | 30.41 | 180 | 5.78 | 341 | 9.85 | 2,146 | 53.96 |  |
| > 51 | 789 | 25.66 | 176 | 5.62 | 462 | 15.53 | 1,785 | 53.19 |  |
|  |  |  |  |  |  |  |  |  |  |
| **Sex** |  |  |  |  |  |  |  |  |  |
| Male | 2,272 | 52.28 | 504 | 12.43 | 793 | 17.8 | 743 | 17.5 | <0.01 |
| Female | 60 | 1.38 | 41 | 0.88 | 175 | 3.6 | 4,568 | 94.15 |  |
|  |  |  |  |  |  |  |  |  |  |
| **Education Level** |  |  |  |  |  |  |  |  |  |
| Primary school and under | 1,123 | 28.86 | 217 | 6.04 | 415 | 9.64 | 2,413 | 55.46 | <0.01 |
| Secondary school | 444 | 25.79 | 126 | 7.7 | 166 | 9.51 | 1,003 | 57 |  |
| High School | 629 | 27.9 | 168 | 7.58 | 279 | 11.86 | 1,335 | 52.66 |  |
| College and postgrad | 136 | 18.08 | 34 | 4.13 | 108 | 13.5 | 557 | 64.28 |  |
|  |  |  |  |  |  |  |  |  |  |
| **Residence** |  |  |  |  |  |  |  |  |  |
| Urban | 964 | 25.33 | 257 | 6.83 | 495 | 12.21 | 2,375 | 55.63 | <0.01 |
| Rural | 1,368 | 28.82 | 288 | 6.4 | 473 | 8.65 | 2,936 | 56.13 |  |
|  |  |  |  |  |  |  |  |  |  |
| **Work Status** |  |  |  |  |  |  |  |  |  |
| Employed | 686 | 37.05 | 154 | 8.28 | 253 | 12.93 | 885 | 41.74 | <0.01 |
| Self-Employed | 1,324 | 41.63 | 221 | 7.48 | 414 | 12.02 | 1,415 | 38.87 |  |
| Non-Labour force | 128 | 4.49 | 103 | 3.7 | 207 | 7.38 | 2,702 | 84.44 |  |
| Unemployed | 194 | 31.7 | 67 | 11.53 | 94 | 13.05 | 306 | 43.72 |  |
|  |  |  |  |  |  |  |  |  |  |
| **Second-hand smoke** |  |  |  |  |  |  |  |  |  |
| Higher | 1,085 | 38.74 | 233 | 8.89 | 335 | 11.48 | 1,210 | 40.89 | <0.01 |
| Lower | 1,247 | 20.39 | 312 | 5.44 | 633 | 10.27 | 4,101 | 63.9 |  |
|  |  |  |  |  |  |  |  |  |  |
| **Knowledge about smoking harm** |  |  |  |  |  |  |  |  |  |
| Higher | 1,141 | 22.67 | 309 | 6.29 | 611 | 11.51 | 3,262 | 59.53 | <0.01 |
| Lower | 1,191 | 33.53 | 236 | 7.22 | 357 | 9.38 | 2,049 | 49.87 |  |
|  |  |  |  |  |  |  |  |  |  |
| **Among current smokers only** |  |  |  |  |  |  |  |  |  |
| trying to stop smoking in the last 12 months |  |  |  |  |  |  |  |  |  |
| yes | 833 | 71.15 | 325 | 28.85 |  |  |  |  | <0.01 |
| no | 1,498 | 86.88 | 218 | 13.12 |  |  |  |  |  |
|  |  |  |  |  |  |  |  |  |  |
| **Thoughts of Quitting** |  |  |  |  |  |  |  |  |  |
| Yes | 1,340 | 76.16 | 398 | 23.84 |  |  |  |  | <0.01 |
| No | 992 | 86.99 | 147 | 13.01 |  |  |  |  |  |
|  |  |  |  |  |  |  |  |  |  |
| **Among daily smokers only** |  |  |  |  |  |  |  |  |  |
| smoking dependency |  |  |  |  |  |  |  |  |  |
| higher | 1,374 | 59.06^‡^ |  |  |  |  |  |  |  |
| lower | 956 | 40.94^‡^ |  |  |  |  |  |  |  |
|  |  |  |  |  |  |  |  |  |  |
| **Smoking level** |  |  |  |  |  |  |  |  |  |
| Heavy | 811 | 33.56^‡^ |  |  |  |  |  |  |  |
| Light | 1,521 | 66.44^‡^ |  |  |  |  |  |  |  |
| * Significance at p < 0.05  ^‡^ Column percentage | | | | | | | | | |

**Supplementary 6. Description of the Global Adult Tobacco Survey Questionnaire.**

1. Household questionnaire

The household questionnaire collected data on all regular residents within the sampled households to determine eligible individuals and record their basic details, facilitating the random selection of a respondent for the individual questionnaire. Basic information, such as age and gender, was collected for all household members. The age data were specifically used to identify an eligible random respondent for the individual questionnaire. Additionally, the questionnaire included questions about the current use of both smoked and smokeless tobacco.

1. Individual Questionnaire

The individual questionnaire collected data from selected individuals who were 15 years old and above. This questionnaire was divided into the following 10 sections:

1. Background characteristics: Questions on sex, age, date of birth, highest education level obtained by respondents, employment status and ownership of household items.
2. Tobacco smoking: Questions addressed tobacco usage patterns (daily, occasional, or non-use), previous tobacco use, age when daily smoking began, types of tobacco products used (such as cigarettes, kretek cigarettes, pipes, and cigars), nicotine dependence, and frequency of attempts to quit.
3. Smokeless tobacco: Questions about usage patterns (daily consumption, less than daily consumption, not at all), and former user of smokeless tobacco
4. Electronic cigarettes: Questions covering patterns of use (daily consumption, less than daily consumption, not at all) of electronic cigarettes.
5. Cessation: Questions regarding advice from healthcare providers to quit smoking, methods attempted to stop smoking, and considerations about quitting smoking.
6. Second-hand smoke: Questions about smoking policy at home, exposure to second-hand smoke at home, workplace indoor smoking policies, and exposure in the past 30 days at workplaces, government buildings, educational institutions, religious facilities, healthcare facilities, restaurants, bars, nightclubs, and on public transportation.
7. Economics—manufactured white cigarettes: Questions regarding the type and quantity of manufactured white cigarette products purchased, their cost, brand, type of product and source of manufactured white cigarette products.
8. Economics—kretek cigarettes: Questions about the type and quantity of kretek cigarettes purchased, their cost, brand, type of product and source of kretek cigarette products.
9. Media: regarding exposure to advertising across various mediums such as television, radio, billboards, posters, newspapers/magazines, cinema, internet, public transportation, public walls, and others were made. This also includes exposure to tobacco-related elements in sporting events, music, theatre, art, or fashion events, as well as participation in tobacco promotion activities. Additionally, respondents' reactions to health warning labels on cigarette packages and their exposure to anti-tobacco advertising and information were assessed. These questions were posed for both white manufactured cigarettes and kretek cigarettes. Media also includes the anti-tobacco media exposure across different media types: newspapers or in magazines, television, radio, billboards, internet, and somewhere else.
10. Knowledge, attitudes and perceptions: Questions regarding knowledge about the health effects of using both smoked and smokeless tobacco.

The household and individual surveys were formulated using the core GATS questionnaire along with additional optional questions tailored for countries undertaking GATS. These questionnaires were adjusted and customized to address pertinent issues relevant to the Indonesian context, with input from the National Health Research and Development Agency, BPS-Statistics Indonesia, WHO Country Office, and the Technical Committee under the Ministry of Health. The modified questionnaires underwent approval by a questionnaire review committee (QRC). Initially crafted in English, they were subsequently translated into Indonesian. Following this translation process, the questionnaires were then back translated into English to ensure translation quality before being utilised for pilot study and actual interview. The implementation of the GATS survey in Indonesia employs the introduction of electronic data collection methods for both household and individual questionnaires, marking a significant advancement in survey methodologies within the country.

**Supplementary 7.Stata Commands**

svydescribe

*GATS 2011*

*dependent var:

//smoking status = SMOKING_STATUS2

rename var442 SMOKINGSTATUS2

tab SMOKINGSTATUS2

*indepandent var

//age group = AGEGROUP

recode AGE 15/18=1 19/30=2 31/50=3 51/99=4, gen(AGEGROUP)

tab AGEGROUP

//sex = SEX

rename A01 SEX

tab SEX

//education level = EDU_CAT

recode A04 1/2=1 3=2 4=3 5/6=4, gen(EDU_CAT)

tab EDU_CAT

//residence = RESIDENCE

tab RESIDENCE

//Work status = WORKSTATUS2

recode A05 1/2=1 3=2 4/6=3 7/8=4 gen(WORKSTATUS2)

tab WORKSTATUS2

//Family members who smoke = FAM_SMOKING

rename HH4E FAM_SMOKING

tab FAM_SMOKING

//smoking normalisation = NORMALISATION2

tab NORMALISATION2

//knowledge about smoking harm = KNOWLEDGE

tab KNOWLEDGE

label define KNOWLEDGE 1"lower" 2"higher"

//antismoking messages = ANTISMOKINGMEDIA

tab var444

rename var444 ANTISMEDIA

tab ANTIMEDIA

//pro-smoking messages= TAPS2

tab TAPS2

*Among current smokers*

//trying to stop smoking in 12-months = TRYINGTOSTOP

rename D01 TRYINGTOSTOP

tab TRYINGTO_STOPSTOP

recode TRYINGTO_STOP 1=1 2=0, gen(TRYING_TOSTOP)

tab TRYING_TOSTOP

//thoughts of quitting smoking

rename D08 THOUGHTSOFQUITTING

tab THOUGHTS_OFQUITTING

recode THOUGHTSOFQUITTING 1/3=1 4=2 -7=2 -9=2, gen(THOUGHTS_2)

tab THOUGHTS_2

//smoking dependency = DEPENDENCY

tab DEPENDENCY

//smoking level = SMOKING_LEVEL

tab SMOKING_LEVEL

*DESCRIPTIVE CHARACTERISTICS*

//complex sample weighting

svyset gatscluster, strata(gatsstrata) weight(gatsweight) vce(linearized) singleunit(missing)

//proportion of each var

svy linearized : proportion SMOKING_STATUS AGEGROUP SEX EDU_CAT RESIDENCE WORKSTATUS2 FAM_SMOKING NORMALISATION2 KNOWLEDGE ANTISMOKING_MEDIA TAPS2 TRYINGTO_STOP THOUGHTS_OFQUITTING FIRST_TRY_SMOKING START_SMOKING_DAILY DEPENDENCY SMOKING_LEVEL

*GATS 2021*

*dependent var:

//smoking status = SMOKING_STATUS2

rename var442 SMOKINGSTATUS2

tab SMOKINGSTATUS2

*indepandent var

//age group = AGEGROUP

recode AGE 15/18=1 19/30=2 31/50=3 51/99=4, gen(AGEGROUP)

tab AGEGROUP

//sex = SEX

rename A01 SEX

tab SEX

//education level = EDU_CAT

recode A04 1/2=1 3=2 4=3 5/6=4, gen(EDU_CAT)

tab EDU_CAT

//residence = RESIDENCE

tab RESIDENCE

//Work status = WORKSTATUS2

recode A05 1/2=1 3=2 4/6=3 7/8=4 gen(WORKSTATUS2)

tab WORKSTATUS2

//Family members who smoke = FAM_SMOKING

rename HH4E FAM_SMOKING

tab FAM_SMOKING

//smoking normalisation = NORMALISATION2

tab NORMALISATION2

//knowledge about smoking harm = KNOWLEDGE

tab KNOWLEDGE

label define KNOWLEDGE 1"lower" 2"higher"

//antismoking messages = ANTISMOKINGMEDIA

tab var444

rename var444 ANTISMEDIA

tab ANTIMEDIA

//pro-smoking messages= TAPS2

tab TAPS2

*Among current smokers*

//trying to stop smoking in 12-months = TRYINGTOSTOP

rename D01 TRYINGTOSTOP

tab TRYINGTO_STOPSTOP

recode TRYINGTO_STOP 1=1 2=0, gen(TRYING_TOSTOP)

tab TRYING_TOSTOP

//thoughts of quitting smoking

rename D08 THOUGHTSOFQUITTING

tab THOUGHTS_OFQUITTING

recode THOUGHTSOFQUITTING 1/3=1 4=2 -7=2 -9=2, gen(THOUGHTS_2)

tab THOUGHTS_2

//smoking dependency = DEPENDENCY

tab DEPENDENCY

//smoking level = SMOKING_LEVEL

tab SMOKING_LEVEL

*DESCRIPTIVE CHARACTERISTICS*

//complex sample weighting

svyset gatscluster, strata(gatsstrata) weight(gatsweight) vce(linearized) singleunit(missing)

//proportion of each var

svy linearized : proportion SMOKING_STATUS AGEGROUP SEX EDU_CAT RESIDENCE WORKSTATUS2 FAM_SMOKING NORMALISATION2 KNOWLEDGE ANTISMOKING_MEDIA TAPS2 TRYINGTO_STOP THOUGHTS_OFQUITTING FIRST_TRY_SMOKING START_SMOKING_DAILY DEPENDENCY SMOKING_LEVEL

//bar chart

svy: mean white if AGEGROUP==1

svy: mean white if AGEGROUP==2

svy: mean white if AGEGROUP==3

svy: mean white if AGEGROUP==4

svy: mean ryo if AGEGROUP==1

svy: mean ryo if AGEGROUP==2

svy: mean ryo if AGEGROUP==3

svy: mean ryo if AGEGROUP==4

svy: mean kretek if AGEGROUP==1

svy: mean kretek if AGEGROUP==2

svy: mean kretek if AGEGROUP==3

svy: mean kretek if AGEGROUP==4

epctile kretek, p(50) svy over(AGEGROUP)

//row and column proportion for smoking behaviour

svy linearized : tabulate AGEGROUP SMOKING_STATUS, row

svy linearized : tabulate SEX SMOKING_STATUS, row

svy linearized : tabulate EDU_CAT SMOKING_STATUS, row

svy linearized : tabulate RESIDENCE SMOKING_STATUS, row

svy linearized : tabulate WORKSTATUS2 SMOKING_STATUS, row

svy linearized : tabulate FAM_SMOKING SMOKING_STATUS, row

svy linearized : tabulate NORMALISATION2 SMOKING_STATUS, row

svy linearized : tabulate KNOWLEDGE SMOKING_STATUS, row

svy linearized : tabulate ANTIMEDIA SMOKING_STATUS, row

svy linearized : tabulate TAPS2 SMOKING_STATUS, row

svy linearized : tabulate TRYINGTO_STOP SMOKING_STATUS

svy linearized : tabulate THOUGHTS_OFQUITTING SMOKING_STATUS

svy linearized : tabulate FIRST_TRY_SMOKING SMOKING_STATUS

svy linearized : tabulate START_SMOKING_DAILY SMOKING_STATUS

svy linearized : tabulate DEPENDENCY SMOKING_STATUS

svy linearized : tabulate SMOKING_LEVEL SMOKING_STATUS

svy linearized : tabulate THOUGHTS_2 SMOKING_STATUS

//row and column proportion for quitting intention

svy linearized : tabulate AGEGROUP TRYING_TOSTOP, row

svy linearized : tabulate SEX TRYING_TOSTOP, row

svy linearized : tabulate EDU_CAT TRYING_TOSTOP, row

svy linearized : tabulate RESIDENCE TRYING_TOSTOP, row

svy linearized : tabulate WORKSTATUS2 TRYING_TOSTOP, row

svy linearized : tabulate NORMALISATION2 TRYING_TOSTOP, row

svy linearized : tabulate KNOWLEDGE TRYING_TOSTOP, row

svy linearized : tabulate THOUGHTS_2 TRYING_TOSTOP, row

svy linearized : tabulate ANTIMEDIA TRYING_TOSTOP, row

svy linearized : tabulate TAPS2 TRYING_TOSTOP, row

svy linearized : tabulate THOUGHTS_2 TRYING_TOSTOP, row

svy linearized : tabulate DEPENDENCY TRYING_TOSTOP, row

svy linearized : tabulate SMOKING_LEVEL TRYING_TOSTOP, row

//row and column proportion for thoughts of quitting

svy linearized : tabulate AGEGROUP THOUGHTS_22, row

svy linearized : tabulate SEX THOUGHTS_22, row

svy linearized : tabulate EDU_CAT THOUGHTS_22, row

svy linearized : tabulate RESIDENCE THOUGHTS_22, row

svy linearized : tabulate WORKSTATUS2 THOUGHTS_22, row

svy linearized : tabulate NORMALISATION2 THOUGHTS_22, row

svy linearized : tabulate KNOWLEDGE THOUGHTS_22, row

svy linearized : tabulate ANTIMEDIA THOUGHTS_22, row

svy linearized : tabulate TAPS2 THOUGHTS_22, row

svy linearized : tabulate TRYING_TOSTOP THOUGHTS_22, row

svy linearized : tabulate DEPENDENCY THOUGHTS_22, row

svy linearized : tabulate SMOKING_LEVEL THOUGHTS_22, row

*Crude Odds Ratio*

1.//for smoking status outcome

svy linearized : logistic SMOKINGSTATUS2 i.ANTIMEDIA, asis allbaselevels

svy linearized : logistic SMOKINGSTATUS2 i.TAPS2

svy linearized : logistic SMOKINGSTATUS2 ib.AGEGROUP

svy linearized : logistic SMOKINGSTATUS2 ib2.SEX

svy linearized : logistic SMOKINGSTATUS2 i.EDU_CAT --

svy linearized : logistic SMOKINGSTATUS2 i.RESIDENCE

svy linearized : logistic SMOKINGSTATUS2 ib2.FAM_SMOKING

svy linearized : logistic SMOKINGSTATUS2 i.WORKSTATUS2 --

svy linearized : logistic SMOKINGSTATUS2 ib2.NORMALISATION2

svy linearized : logistic SMOKINGSTATUS2 ib2.KNOWLEDGE

2.//for attempts to quit outcome

svy linearized : logistic TRYING_TOSTOP i.ANTIMEDIA

svy linearized : logistic TRYING_TOSTOP i.TAPS2

svy linearized : logistic TRYING_TOSTOP ib4.AGEGROUP

svy linearized : logistic TRYING_TOSTOP ib2.SEX

svy linearized : logistic TRYING_TOSTOP i.EDU_CAT

svy linearized : logistic TRYING_TOSTOP i.RESIDENCE

svy linearized : logistic TRYING_TOSTOP i.WORKSTATUS2

svy linearized : logistic TRYING_TOSTOP ib2.NORMALISATION2

svy linearized : logistic TRYING_TOSTOP ib2.KNOWLEDGE

svy linearized : logistic TRYING_TOSTOP ib2.THOUGHTS_2

svy linearized : logistic TRYING_TOSTOP ib1.dependency

svy linearized : logistic TRYING_TOSTOP ib1.SMOKING_LEVEL

3.//for thoughts of quitting outcome

svy linearized : logistic THOUGHTS_22 i.ANTIMEDIA

svy linearized : logistic THOUGHTS_22 i.TAPS2

svy linearized : logistic THOUGHTS_22 ib4.AGEGROUP

svy linearized : logistic THOUGHTS_22 ib2.SEX

svy linearized : logistic THOUGHTS_22 i.EDU_CAT

svy linearized : logistic THOUGHTS_22 i.RESIDENCE

svy linearized : logistic THOUGHTS_22 i.WORKSTATUS2

svy linearized : logistic THOUGHTS_22 ib2.NORMALISATION2

svy linearized : logistic THOUGHTS_22 ib2.KNOWLEDGE

svy linearized : logistic THOUGHTS_22 ib2.THOUGHTS_2

svy linearized : logistic THOUGHTS_22 ib1.dependency

svy linearized : logistic THOUGHTS_22 ib1.SMOKING_LEVEL

svy linearized : logistic THOUGHTS_22 ib0.trying_tostop

*adjusted OR

//full model smoking status

svy linearized : logistic SMOKINGSTATUS2 ib1.ANTIMEDIA ib1.TAPS2 ib4.AGEGROUP i.EDU_CAT i.RESIDENCE ib2.NORMALISATION2 ib2.KNOWLEDGE i.WORKSTATUS2, asis allbaselevels

estimate store A

//reduced model smoking status

svy linearized : logistic SMOKINGSTATUS2 ib1.ANTIMEDIA ib1.TAPS2 ib4.AGEGROUP i.EDU_CAT i.RESIDENCE ib2.NORMALISATION2 ib2.KNOWLEDGE, asis allbaselevels

//full model attemps to quit

svy linearized : logistic TRYING_TOSTOP ib1.ANTIMEDIA ib1.TAPS2 ib4.AGEGROUP i.EDU_CAT i.RESIDENCE ib2.NORMALISATION2 ib2.KNOWLEDGE i.WORKSTATUS2 ib2.THOUGHTS_2 ib1.dependency ib1.SMOKING_LEVEL, asis allbaselevels

//full model thoughts of quitting

svy linearized : logistic THOUGHTS_22 ib1.ANTIMEDIA ib1.TAPS2 ib4.AGEGROUP i.EDU_CAT i.RESIDENCE ib2.NORMALISATION2 ib2.KNOWLEDGE i.WORKSTATUS2 ib1.dependency ib1.SMOKING_LEVEL ib0.trying_tostop, asis allbaselevels

*adjusted stratified male and female

xi: svy: logistic SMOKINGSTATUS2 i.ANTIMEDIA i.TAPS2 ib4.AGEGROUP i.EDU_CAT ib1.RESIDENCE i.WORKSTATUS2 ib2.NORMALISATION2 ib2.KNOWLEDGE if SEX==1

xi: svy: logistic SMOKINGSTATUS2 i.ANTIMEDIA i.TAPS2 ib4.AGEGROUP i.EDU_CAT ib1.RESIDENCE i.WORKSTATUS2 ib2.NORMALISATION2 ib2.KNOWLEDGE if SEX==2

xi: svy: logistic TRYING_TOSTOP i.ANTIMEDIA i.TAPS2 ib4.AGEGROUP i.EDU_CAT ib2.RESIDENCE i.WORKSTATUS2 i.NORMALISATION2 ib2.KNOWLEDGE ib2.THOUGHTS_2 ib1.dependency ib1.SMOKING_LEVEL if SEX==1

xi: svy: logistic TRYING_TOSTOP i.ANTIMEDIA i.TAPS2 i.EDU_CAT ib2.RESIDENCE ib2.KNOWLEDGE ib2.THOUGHTS_2 if SEX==2

xi: svy: logistic SMOKINGSTATUS2 ib1.ANTIMEDIA ib1.TAPS2 ib4.AGEGROUP i.EDU_CAT i.RESIDENCE ib2.NORMALISATION2 ib2.KNOWLEDGE i.WORKSTATUS2 if SEX==1

xi: svy: logistic SMOKINGSTATUS2 ib1.ANTIMEDIA ib1.TAPS2 ib4.AGEGROUP i.EDU_CAT i.RESIDENCE ib2.NORMALISATION2 ib2.KNOWLEDGE i.WORKSTATUS2 if SEX==2

xi: svy: logistic TRYING_TOSTOP ib1.ANTIMEDIA ib1.TAPS2 ib4.AGEGROUP i.EDU_CAT ib2.RESIDENCE ib2.NORMALISATION2 ib2.KNOWLEDGE i.WORKSTATUS2 ib2.THOUGHTS_2 ib1.dependency ib1.SMOKING_LEVEL if SEX==1

xi: logistic TRYING_TOSTOP ib1.ANTIMEDIA ib1.TAPS2 ib4.AGEGROUP i.EDU_CAT ib2.RESIDENCE ib2.NORMALISATION2 ib2.KNOWLEDGE i.WORKSTATUS2 ib2.THOUGHTS_2 ib1.dependency ib1.SMOKING_LEVEL if SEX==2 [iweight=gatsweight]

xi: logistic trying_tostop ib1.ANTIMEDIA ib1.TAPS2 ib2.RESIDENCE ib2.NORMALISATION2 ib2.KNOWLEDGE ib2.THOUGHTS_2 ib1.dependency ib1.SMOKING_LEVEL if SEX==2 [iweight=gatsweight]

xi: logistic TRYING_TOSTOP ib1.ANTIMEDIA ib1.TAPS2 ib4.AGEGROUP i.EDU_CAT ib2.RESIDENCE ib2.NORMALISATION2 ib2.KNOWLEDGE i.WORKSTATUS2 ib2.THOUGHTS_2 ib1.dependency ib1.SMOKING_LEVEL if SEX==2 [iweight=gatsweight]

xi: svy: logistic THOUGHTS_22 ib1.ANTIMEDIA ib1.TAPS2 ib4.AGEGROUP i.EDU_CAT ib2.RESIDENCE ib2.NORMALISATION2 ib2.KNOWLEDGE i.WORKSTATUS2 ib1.dependency ib1.SMOKING_LEVEL ib0.trying_tostop if SEX==1

xi: logistic THOUGHTS_22 ib1.ANTIMEDIA ib1.TAPS2 ib4.AGEGROUP i.EDU_CAT ib2.RESIDENCE ib2.NORMALISATION2 ib2.KNOWLEDGE i.WORKSTATUS2 ib1.dependency ib1.SMOKING_LEVEL if SEX==2 [iweight=gatsweight]

xi: logistic THOUGHTS_22 ib1.ANTIMEDIA ib1.TAPS2 i.EDU_CAT ib2.RESIDENCE ib2.NORMALISATION2 ib2.KNOWLEDGE ib1.dependency ib1.SMOKING_LEVEL if SEX==2 [iweight=gatsweight]

*Stratification of male & female being current smoker*

1.//by AGEGROUP

xi: svy: logistic SMOKINGSTATUS2 i.AGEGROUP ib2.SEX

xi: svy: logistic SMOKINGSTATUS2 i.AGEGROUP ib1.SEX

xi: svy: logistic SMOKINGSTATUS2 ib4.AGEGROUP if SEX==1

xi: svy: logistic SMOKINGSTATUS2 ib4.AGEGROUP if SEX==2

2.//by education level

xi: svy: logistic SMOKINGSTATUS2 i.EDU_CAT ib2.SEX

xi: svy: logistic SMOKINGSTATUS2 i.EDU_CAT i.SEX

xi: svy: logistic SMOKINGSTATUS2 i.EDU_CAT if SEX==1

xi: svy: logistic SMOKINGSTATUS2 i.EDU_CAT if SEX==2

3.//by residence

xi: svy: logistic SMOKINGSTATUS2 ib1.RESIDENCE ib2.SEX

xi: svy: logistic SMOKINGSTATUS2 ib1.RESIDENCE ib1.SEX

xi: svy: logistic SMOKINGSTATUS2 ib1.RESIDENCE if SEX==1

xi: svy: logistic SMOKINGSTATUS2 ib1.RESIDENCE if SEX==2

4.//by workstatus

xi: svy: logistic SMOKINGSTATUS2 i.WORKSTATUS2 ib2.SEX

xi: svy: logistic SMOKINGSTATUS2 i.WORKSTATUS2 ib1.SEX

xi: svy: logistic SMOKINGSTATUS2 i.WORKSTATUS2 if SEX==1

xi: svy: logistic SMOKINGSTATUS2 i.WORKSTATUS2 if SEX==2

5.//by smoking normalisation/secondhand smoke

xi: svy: logistic SMOKINGSTATUS2 ib2.NORMALISATION2 ib2.SEX

xi: svy: logistic SMOKINGSTATUS2 ib2.NORMALISATION2 ib1.SEX

xi: svy: logistic SMOKINGSTATUS2 ib2.NORMALISATION2 if SEX==1

xi: svy: logistic SMOKINGSTATUS2 ib2.NORMALISATION2 if SEX==2

6.//by knowledge

xi: svy: logistic SMOKINGSTATUS2 ib2.KNOWLEDGE ib2.SEX

xi: svy: logistic SMOKINGSTATUS2 ib2.KNOWLEDGE ib1.SEX

xi: svy: logistic SMOKINGSTATUS2 ib2.KNOWLEDGE if SEX==1

xi: svy: logistic SMOKINGSTATUS2 ib2.KNOWLEDGE if SEX==2

7.// by antismoking media

xi: svy: logistic SMOKINGSTATUS2 ib1.ANTIMEDIA ib2.SEX

xi: svy: logistic SMOKINGSTATUS2 ib1.ANTIMEDIA ib2.SEX

xi: svy: logistic SMOKINGSTATUS2 ib1.ANTIMEDIA if SEX==1

xi: svy: logistic SMOKINGSTATUS2 ib1.ANTIMEDIA if SEX==2

8.// by cigarette advertisements, promotions, and sponsorships

xi: svy: logistic SMOKINGSTATUS2 ib1.TAPS2 ib2.SEX

xi: svy: logistic SMOKINGSTATUS2 ib1.TAPS2 ib1.SEX

xi: svy: logistic SMOKINGSTATUS2 ib1.TAPS2 if SEX==1

xi: svy: logistic SMOKINGSTATUS2 ib1.TAPS2 if SEX==2

*Stratification of male & female having attempt to quit smoking*

1.//by AGEGROUP

xi: svy: logistic TRYING_TOSTOP ib4.AGEGROUP if SEX==1

xi: logistic TRYING_TOSTOP ib4.AGEGROUP if SEX==2 [iweight=gatsweight]

table ( AGEGROUP ) ( TRYING_TOSTOP ) ( SEX )

2.//by education level

xi: svy: logistic TRYING_TOSTOP i.EDU_CAT if SEX==1

xi: svy: logistic TRYING_TOSTOP i.EDU_CAT if SEX==2

xi: logistic TRYING_TOSTOP i.EDU_CAT if SEX==2 [iweight=gatsweight]

3.//by residence

xi: svy: logistic TRYING_TOSTOP ib2.RESIDENCE if SEX==1

xi: logistic TRYING_TOSTOP ib2.RESIDENCE if SEX==2 [iweight=gatsweight]

4.//by workstatus

xi: svy: logistic TRYING_TOSTOP i.WORKSTATUS2 if SEX==1

xi: logistic TRYING_TOSTOP i.WORKSTATUS2 if SEX==2 [iweight=gatsweight]

5.//by smoking secondhandsmoke

xi: svy: logistic TRYING_TOSTOP ib2.NORMALISATION2 if SEX==1

xi: logistic TRYING_TOSTOP ib2.NORMALISATION2 if SEX==2 [iweight=gatsweight]

6.//by knowledge

xi: svy: logistic TRYING_TOSTOP ib2.KNOWLEDGE if SEX==1

xi: logistic TRYING_TOSTOP ib2.KNOWLEDGE if SEX==2 [iweight=gatsweight]

7.//by thoughts of quitting

xi: svy: logistic TRYING_TOSTOP ib2.THOUGHTS_2 if SEX==1

xi: logistic TRYING_TOSTOP ib2.THOUGHTS_2 if SEX==2 [iweight=gatsweight]

8.// by smoking dependency

xi: svy: logistic TRYING_TOSTOP ib1.dependency if SEX==1

xi: logistic TRYING_TOSTOP ib1.dependency if SEX==2 [iweight=gatsweight]

9.// by smoking level

xi: svy: logistic TRYING_TOSTOP ib1.SMOKING_LEVEL if SEX==1

xi: logistic TRYING_TOSTOP ib1.SMOKING_LEVEL if SEX==2 [iweight=gatsweight]

7.// by antismoking media

xi: svy: logistic TRYING_TOSTOP i.ANTIMEDIA ib2.SEX

xi: svy: logistic TRYING_TOSTOP i.ANTIMEDIA i.SEX

xi: svy: logistic TRYING_TOSTOP ib1.ANTIMEDIA if SEX==1

xi: logistic TRYING_TOSTOP ib1.ANTIMEDIA if SEX==2 [iweight=gatsweight]

8.// by cigarette advertisements, promotions, and sponsorships

xi: svy: logistic TRYING_TOSTOP i.TAPS2 ib2.SEX

xi: svy: logistic TRYING_TOSTOP i.TAPS2 i.SEX

xi: svy: logistic TRYING_TOSTOP ib1.TAPS2 if SEX==1

xi: logistic TRYING_TOSTOP ib1.TAPS2 if SEX==2 [iweight=gatsweight]

*Stratification of male & female having thoughts to quit smoking*

recode THOUGHTS_2 1=1 2=0, gen(THOUGHTS_22)

tab THOUGHTS_2

tab THOUGHTS_22

1.//by AGEGROUP

xi: svy: logistic THOUGHTS_22 ib4.AGEGROUP if SEX==1

xi: logistic THOUGHTS_22 ib4.AGEGROUP if SEX==2 [iweight=gatsweight]

2.//by education level

xi: svy: logistic THOUGHTS_22 i.EDU_CAT if SEX==1

xi: logistic THOUGHTS_22 i.EDU_CAT if SEX==2 [iweight=gatsweight]

3.//by residence

xi: svy: logistic THOUGHTS_22 ib2.RESIDENCE if SEX==1

xi: logistic THOUGHTS_22 ib2.RESIDENCE if SEX==2 [iweight=gatsweight]

4.//by workstatus

xi: svy: logistic THOUGHTS_22 i.WORKSTATUS2 if SEX==1

xi: logistic THOUGHTS_22 i.WORKSTATUS2 if SEX==2 [iweight=gatsweight]

5.//by smoking normalisation

xi: svy: logistic THOUGHTS_22 ib2.NORMALISATION2 if SEX==1

xi: logistic THOUGHTS_22 ib2.NORMALISATION2 if SEX==2 [iweight=gatsweight]

6.//by knowledge

xi: svy: logistic THOUGHTS_22 ib2.KNOWLEDGE if SEX==1

xi: logistic THOUGHTS_22 ib2.KNOWLEDGE if SEX==2 [iweight=gatsweight]

7.// by smoking dependency

xi: svy: logistic THOUGHTS_22 ib1.dependency if SEX==1

xi: logistic THOUGHTS_22 ib1.dependency if SEX==2 [iweight=gatsweight]

8.// by smoking level

xi: svy: logistic THOUGHTS_22 ib1.SMOKING_LEVEL if SEX==1

xi: logistic THOUGHTS_22 ib1.SMOKING_LEVEL if SEX==2 [iweight=gatsweight]

9.// by antismoking media

xi: svy: logistic THOUGHTS_22 ib1.ANTIMEDIA if SEX==1

xi: logistic THOUGHTS_22 ib1.ANTIMEDIA if SEX==2 [iweight=gatsweight]

10.// by cigarette advertisements, promotions, and sponsorships

xi: svy: logistic THOUGHTS_22 ib1.TAPS2 if SEX==1

xi: logistic THOUGHTS_22 ib1.TAPS2 if SEX==2 [iweight=gatsweight]

**Bibliography**

1. Global Adult Tobacco Survey Collaborative Group. Global Adult Tobacco Survey (GATS): Core Questionnaire with Optional Questions [Internet]. Atlanta: Centers for Disease Control and Prevention; 2020 [cited 2024 21 April 2024]. Available from: https://nccd.cdc.gov/GTSSDataSurveyResources/Ancillary/DataReports.aspx?CAID=2&Survey=4&WHORegion=2&Country=181&Site=385000
2. The Ministry of Health of Republic Indonesia. Global Adult Tobacco Survey: Indonesia Report 2011 [Internet]. Jakarta: The Ministry of Health of Republic Indonesia; 2012 [cited 2024 21 April 2024]. Available from: https://cdn.who.int/media/docs/default-source/ncds/ncd-surveillance/data-reporting/indonesia/gats/2011-gats-indonesia-report.pdf?sfvrsn=ba5130b7_2
